# Importance of the renal ion channel TRPM6 in the circadian secretion of renin to raise blood pressure

**DOI:** 10.1038/s41467-021-24063-2

**Published:** 2021-06-17

**Authors:** Yosuke Funato, Daisuke Yamazaki, Daisuke Okuzaki, Nobuhiko Yamamoto, Hiroaki Miki

**Affiliations:** 1 Department of Cellular Regulation, Research Institute for Microbial Diseases, Osaka University, Osaka, Japan; 2 Genome Information Research Center, Research Institute for Microbial Diseases, Osaka University, Osaka, Japan; 3 Neuroscience Laboratories, Graduate School of Frontier Biosciences, Osaka University, Osaka, Japan

**Keywords:** Cell biology, Physiology, Hypertension, Kidney

## Abstract

Blood pressure has a daily pattern, with higher values in the active period. Its elevation at the onset of the active period substantially increases the risk of fatal cardiovascular events. Renin secretion stimulated by renal sympathetic neurons is considered essential to this process; however, its regulatory mechanism remains largely unknown. Here, we show the importance of transient receptor potential melastatin-related 6 (TRPM6), a Mg^2+^-permeable cation channel, in augmenting renin secretion in the active period. TRPM6 expression is significantly reduced in the distal convoluted tubule of hypotensive *Cnnm2*-deficient mice. We generate kidney-specific *Trpm6*-deficient mice and observe a decrease in blood pressure and a disappearance of its circadian variation. Consistently, renin secretion is not augmented in the active period. Furthermore, renin secretion after pharmacological activation of β-adrenoreceptor, the target of neuronal stimulation, is abrogated, and the receptor expression is decreased in renin-secreting cells. These results indicate crucial roles of TRPM6 in the circadian regulation of blood pressure.

## Abstract (web summary)

Circadian variation of blood pressure, with higher values in the active period, is associated with the risk of fatal cardiovascular events. Here, we show the importance of renal TRPM6, a Magnesium-permeable cation channel, in raising blood pressure by stimulating renin secretion.

## Introduction

Approximately one billion people worldwide are estimated to have hypertension, which significantly increases the risk of various diseases, including potentially fatal diseases, such as ischemic heart disease and stroke. These hypertension-related fatal incidents are known to occur frequently in the early morning, the beginning of the active period when blood pressure rises sharply^1^. Renin secretion triggered by renal sympathetic neurons is considered to play a crucial role in this blood pressure elevation, but its regulatory mechanism is not well understood^2^.

It is widely accepted that some dietary minerals, particularly sodium and potassium, play important roles in the control of blood pressure. Magnesium is a major essential element involved in a variety of biological activities. Epidemiological studies have shown a significant inverse relationship between dietary magnesium levels and the risk of hypertension^3–6^. Moreover, urinary magnesium excretion, which is assumed to be in approximate equilibrium with intestinal magnesium absorption, is inversely correlated with the risk of hypertension^7^. These findings suggest a close association between organismal magnesium homeostasis and blood pressure regulation, highlighting the importance of Mg^2+^ channels and/or transporters in the regulation of blood pressure.

A large quantity of magnesium is constantly being reabsorbed in the kidney. Most magnesium in the glomerular filtrate is reabsorbed in the thick ascending limb of Henle’s loop, but the final step of reabsorption occurs at the distal convoluted tubule (DCT)^8^. The latter process is tightly regulated to adjust the amount of reabsorption to a level appropriate for maintaining magnesium homeostasis. Genomic analyses of congenital diseases with symptoms of hypomagnesemia have revealed several key molecules involved in this process. *Trpm6*, encoding transient receptor potential melastatin-related 6 (TRPM6), is mutated in patients with recessive hypomagnesemia with secondary hypocalcemia^9,10^. TRPM6 forms a Mg^2+^-permeable ion channel localized to the apical membrane of DCT cells and mediates Mg^2+^ intake from the tubular lumen^11^. Another key molecule is cyclin M2 (CNNM2), a Mg^2+^ transporter localized to the basolateral membrane of DCT cells^12,13^. *Cnnm2* mutations cause familial dominant hypomagnesemia, which is characterized by defects in magnesium reabsorption in the kidney^12^. *Cnnm2*-deficient mice show similar symptoms of hypomagnesemia with magnesium wasting in the kidney^14^. The precise molecular role of CNNM2 is still controversial, but several lines of evidence suggest that it mediates Mg^2+^ efflux from DCT cells through the basolateral membrane^15^, thereby contributing to magnesium reabsorption. Further analyses of *Cnnm2*-deficient mice have revealed that these mice also have significantly lower blood pressure than control mice^14^. However, the mechanistic details and the involvement of other Mg^2+^ channels/transporters in this blood pressure regulation remain unknown.

The renal DCT is also known to play critical roles in blood pressure regulation, because it significantly reabsorbs sodium and regulates body fluid volume^16^. A number of cation channels/transporters, such as the Na-Cl co-transporter (NCC), epithelial sodium channel (ENaC), and Na^+^/H^+^ exchanger 2 (NHE2), are expressed at the DCT apical membrane and some of these proteins mediate Na^+^ intake into DCT cells from the tubular lumen. Intracellular Na^+^ is then extruded from the cells through the basolateral membrane by Na^+^/K^+^-ATPase to accomplish its reabsorption. Apart from a direct involvement in regulating body fluid volume, the DCT is located adjacent to the macula densa, which controls secretion of renin, a hormone with the principal role in orchestrating blood pressure regulation^17,18^. By sensing urinary Cl^−^ levels, macula densa cells stimulate nearby juxtaglomerular (JG) cells to secrete renin.

In this work, we perform transcriptome analyses with kidneys of hypotensive *Cnnm2*-deficient mice. We find a significant decrease of *Trpm6*, prompting us to generate kidney-specific *Trpm6*-deficient mice. Unexpectedly, analyses of this mouse strain reveal a phenotype qualitatively different from that of *Cnnm2*-deficient: the loss of circadian variation of blood pressure. We also find that this phenotype is caused by impaired secretion of renin, a blood pressure-raising hormone of which blood level is normally elevated during the active phase.

## Results

### Downregulation of TRPM6 expression in *Cnnm2*-deficient mice

To explore the molecular mechanism of blood pressure reduction in *Cnnm2*-deficient mice, we performed DNA microarray analyses to investigate gene expression changes in the kidney of *Cnnm2*^fl/fl^;*Six2-Cre* mice, lacking both *Cnnm2* alleles in the kidney^14^. Among the differentially expressed genes (Table 1), we chose to analyze *Pvalb* and *Trpm6*, both of which are expressed in the DCT^9,19^. Quantitative PCR (qPCR) analyses revealed that *Trpm6* expression was similarly reduced in the kidneys of both *Cnnm2*^fl/fl^;*Six2-Cre* and *Cnnm2*^+/Δ^ mice (Supplementary Fig. 1a). Since both *Cnnm2*^+/Δ^ and *Cnnm2*^fl/fl^;*Six2-Cre* mice were hypotensive^14^, we analyzed TRPM6 expression in more detail. Immunoblotting analyses of kidney lysates confirmed a significant decrease of TRPM6 at the protein level by *Cnnm2*-deficiency, while the levels of NCC and phosphorylated NCC (active form) were not markedly affected (Supplementary Fig. 1b, CNNM2 expression was confirmed by successive immunoprecipitation and immunoblotting analyses). The decrease of TRPM6 in the kidneys of *Cnnm2*^fl/fl^;*Six2-Cre* mice was also confirmed by immunofluorescence analyses (Supplementary Fig. 1c).

**Table 1:** Genes that were upregulated or downregulated in the kidneys of *Cnnm2*^fl/fl^;*Six2-Cre* mice.

Fold	*p-*value	Gene symbol	Gene name
6.394	0.046	Cldn11	Claudin 11
5.002	0.016	Slc14a2	Solute carrier family 14 (urea transporter), member 2, transcript variant 2
4.109	0.066	Pde4d	Phosphodiesterase 4D
3.834	0.026	Aldh1a3	Aldehyde dehydrogenase family 1, subfamily A3
3.379	0.050	Gcnt3	Glucosaminyl (N-acetyl) transferase 3, mucin type
2.872	0.090	Tal2	T-cell acute lymphocytic leukemia 2
2.812	0.091	Gm567	Predicted gene 567
2.758	0.049	1810037I17Rik	RIKEN cDNA 1810037I17 gene
2.724	0.026	AA467197	AA467197
2.467	0.029	Crlf1	Cytokine receptor-like factor 1
2.446	0.015	Calml3	Calmodulin-like 3
2.445	0.081	Gulo	Gulonolactone (L-) oxidase
2.401	0.067	Akr1b3	Aldo-keto reductase family 1, member B3 (aldose reductase)
2.389	0.074	Adh6a	Alcohol dehydrogenase 6 A (class V)
2.234	0.006	Fgb	Fibrinogen beta chain
2.203	0.043	Ndrg4	N-myc downstream regulated gene 4, transcript variant A
2.126	0.026	R3hdml	R3H domain containing-like
2.091	0.014	Lamb3	Laminin, beta 3, transcript variant 1
2.024	0.012	Gm3916	Predicted gene 3916, misc_RNA
**−**2.106	0.096	B020031M17Rik	RIKEN cDNA B020031M17 gene
**−**2.292	0.049	Olfr1372-ps1	Olfactory receptor 1372, pseudogene 1, non-coding RNA
**−**2.430	0.002	Grin2c	Glutamate receptor, ionotropic, NMDA2C (epsilon 3)
−2.648	0.077	Trpm6	Transient receptor potential cation channel, subfamily M, member 6
−2.730	0.030	Mapk8	Mitogen-activated protein kinase 8
−3.768	0.099	Cmtm4	CKLF-like MARVEL transmembrane domain containing 4
−4.472	0.062	Pvalb	Parvalbumin
−6.364	0.051	Cnnm2	Cyclin M2, transcript variant 1

Signature genes (*p* < 0.1; two-tailed Student’s *t*-test (paired)) with mean fold-changes > +2.0 (more than twice) or < − 2.0 (less than half) are listed.

We also examined TRPM6 expression in mice lacking CNNM4, which also show symptoms of hypomagnesemia^20^ but have elevated blood pressure^14^. Immunoblotting and immunofluorescence analyses indicated that TRPM6 expression is augmented in DCT cells of *Cnnm4*-deficient mice (Supplementary Fig. 1d, e), suggesting that TRPM6 expression is closely associated with blood pressure values, but it is not directly linked to blood magnesium levels.

### Regulation of TRPM6 expression by intracellular Mg^2+^ in DCT cells

To characterize the molecular mechanism of TRPM6 downregulation in *Cnnm2*-deficient mice, we used a DCT-derived cell line MDCT^21^, which expresses dominantly *Cnnm2* and moderately *Cnnm3* and *Cnnm4* among *Cnnm* family genes (*Cnnm1*–*Cnnm4*, Supplementary Fig. 1f). As CNNM2 and CNNM4 have strong Mg^2+^ efflux activity while CNNM3 has no Mg^2+^ efflux activity^15^, we performed small-interfering RNA (siRNA)-mediated knockdown of either *Cnnm2*, *Cnnm4*, or both. *Cnnm2* knockdown augmented intracellular Mg^2+^ levels ([Mg^2+^]_i_), and simultaneous knockdown of *Cnnm4* led to slight elevation of average [Mg^2+^]_i_ value, which was not statistically significant (Supplementary Fig. 1g). We next examined *Trpm6* expression by qPCR and found a significant decrease after *Cnnm2* knockdown and a much larger decrease after double knockdown of *Cnnm2* and *Cnnm4*. Therefore, *Trpm6* expression appears to be negatively regulated by intracellular Mg^2+^ levels, which is consistent with previous reports^22,23^. To directly examine the importance of Mg^2+^, we cultured MDCT cells in media with various concentrations of Mg^2+^. As shown in Supplementary Fig. 1h, decreasing the extracellular Mg^2+^ concentration led to the reduction in intracellular Mg^2+^ level and the increase of *Trpm6* expression, suggesting a suppressive role of Mg^2+^. In the case of double knockdown (*Cnnm2*/*Cnnm4*) MDCT cells, *Trpm6* expression was suppressed with decreasing Mg^2+^ concentrations down to 0.2 mM, but further reductions canceled the suppressive effect; both intracellular Mg^2+^ and *Trpm6* levels were not significantly different from control siRNA-transfected cells in both 0.1 and 0.02 mM conditions. These results indicate a Mg^2+^-dependent regulatory mechanism of *Trpm6* expression, which explains the *Trpm6* downregulation in the kidneys of *Cnnm2*-deficient mice.

### Conditional disruption of *Trpm6* in the kidney

Next, we aimed to generate *Trpm6*-deficient mice to further investigate the importance TRPM6 in the regulation of blood pressure. We used an embryonic stem (ES) cell clone carrying a recombinant *Trpm6* allele (Trpm6^−^) that contains a splice acceptor sequence, forcing premature mRNA splicing and resulting in an mRNA truncated after exon 6 (Supplementary Fig. 2a). Chimeric heterozygous mice were obtained by blastocyst injection of the ES cells and recombination was confirmed by PCR (Supplementary Fig. 2b). We intercrossed these *Trpm6*^+/−^ mice, but no *Trpm6*^−/−^ offspring were obtained (Supplementary Fig. 2c). This result is consistent with previous reports showing embryonic lethality in *Trpm6*-deficient mice^24,25^.

**Fig. 1:**
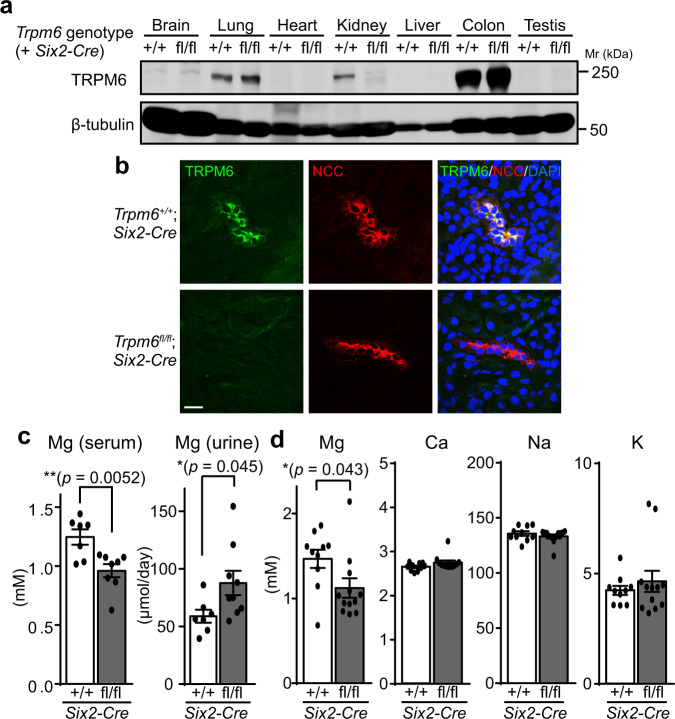
Disrupted magnesium homeostasis in *Trpm6*-deficient mice. **a** Lysates obtained from various organs of 2-month-old *Trpm6*^+/+^;*Six2-Cre* and *Trpm6*^fl/fl^;*Six2-Cre* mice were subjected to immunoblotting with an anti-TRPM6 antibody. A representative result from two independent experiments with similar results are shown. **b** Cryosections of 2-month-old *Trpm6*^+/+^;*Six2-Cre* and *Trpm6*^fl/fl^;*Six2-Cre* mice were subjected to immunofluorescence staining with an anti-TRPM6 antibody (green), an anti-NCC antibody (red), and DAPI (blue). Bar, 20 μm. A representative result from three independent experiments with similar results are shown. **c** Colorimetric quantitation of magnesium (Mg) in serum and urine samples obtained from 2- to 3-month-old mice of *Trpm6*^+/+^;*Six2-Cre* and *Trpm6*^fl/fl^;*Six2-Cre* mice. Data are shown as means ± SEM (from left to right, *n* = 7, 8, 7, 9 mice). The *p*-values were determined by Student’s two-tailed *t*-tests (unpaired). *p <  0.05, **p < 0.01. **d** Serum samples were subjected to elemental analyses using ICP-ES. Data are shown as means ± SEM (*n* = 10 for *Trpm6*^+/+^;*Six2-Cre* mice and *n* = 12 for *Trpm6*^fl/fl^;*Six2-Cre* mice). The *p*-values were determined by Student’s two-tailed *t*-tests (unpaired). *p < 0.05.

Therefore, we performed a kidney-specific conditional disruption of *Trpm6* using *Six2-Cre*, which enables efficient and specific expression of GFP-fused Cre recombinase in the embryonic kidney (ref. ^26^ and Supplementary Fig. 3). *Trpm6*^fl/fl^;*Six2-Cre* mice (kidney-specific *Trpm6*-deficient mice) were born at the expected Mendelian ratio (Supplementary Fig. 2c). To determine the expression of TRPM6, we performed immunoblotting analyses using an anti-TRPM6 antibody. TRPM6 protein levels were greatly reduced in the kidneys of *Trpm6*^fl/fl^;*Six2-Cre* mice, but no significant changes were observed in other organs (Fig. 1a). Immunofluorescence staining analyses also showed no expression of TRPM6 in NCC-positive DCT cells (Fig. 1b). We observed no significant TRPM6-positive signal in more than 100 such clusters of NCC-positive cells, suggesting that TRPM6 expression is almost completely suppressed in DCT cells of *Trpm6*^fl/fl^;*Six2-Cre* mice. These results confirm the successful generation of kidney-specific *Trpm6*-deficient mice.

We first examined the effect of kidney-specific *Trpm6*-deficiency on magnesium homeostasis. We maintained mice in metabolic cages and collected urine and serum samples, which were then subjected to colorimetric quantitation of magnesium levels using Xylidyl Blue-I. Serum magnesium levels were significantly lower in *Trpm6*^fl/fl^;*Six2-Cre* mice compared to those in control *Trpm6*^+/+^;*Six2-Cre* mice, whereas urinary magnesium levels were higher (Fig. 1c). We also performed inductively coupled plasma-emission spectroscopy (ICP-ES) analyses to measure several major metal elements in the serum and found that only magnesium levels were significantly affected in *Trpm6*^fl/fl^;*Six2-Cre* mice (Fig. 1d). These results clearly indicate that kidney-specific *Trpm6*-deficient mice have hypomagnesemia with renal magnesium wasting, as observed in kidney-specific *Cnnm2*-deficient mice^14^.

### Lack of circadian variation of blood pressure and renin activity

**Fig. 2:**
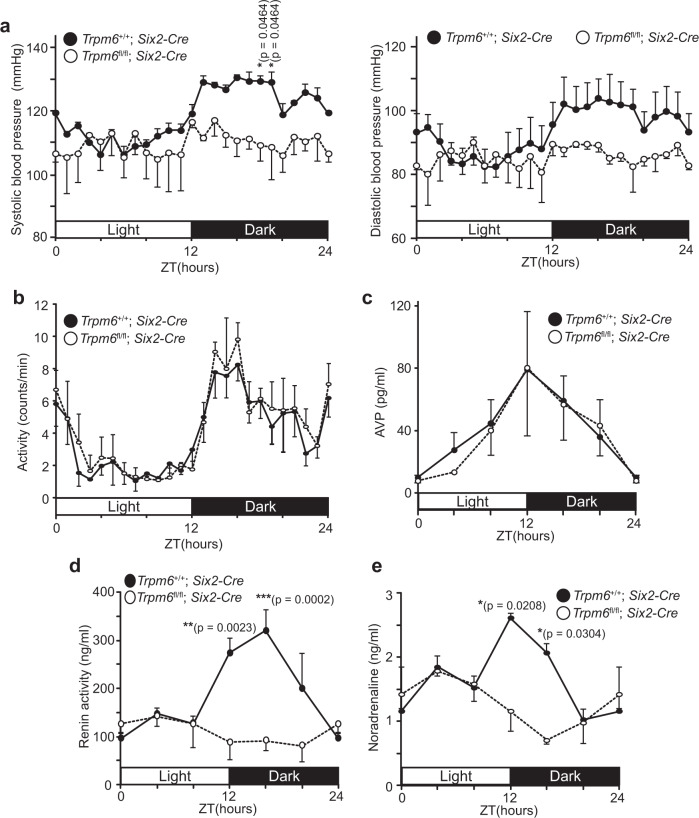
*Trpm6* ablation impairs circadian rhythms of blood pressure and renin activity. **a** Blood pressure values of 2- to 3-month-old mice of the indicated genotypes were measured with radiotelemetry. Data are shown as means ± SEM for systolic (left) and diastolic (right) pressure values (*n* = 4 for *Trpm6*^+/+^; *Six2-Cre* and *n* = 3 for *Trpm6*^fl/fl^; *Six2-Cre*). The *p*-values were determined by two-way ANOVA with Holm-Sidak post hoc tests. *p < 0.05. **b** Locomotor activity of 2- to 3-month-old mice of the indicated genotypes. Data are shown as means ± SEM (*n* = 3 mice for each genotype). **c**–**e** Whole blood was collected from 2- to 4-month-old mice of the indicated genotypes at indicated time periods, and plasma AVP (**c**, *n* = 3 mice for each genotype), renin activity (**d**, *n* = 5 at ZT0 and ZT4, *n* = 6 for *Trpm6*^+/+^; *Six2-Cre* and *n* = 4 for *Trpm6*^fl/fl^; *Six2-Cre* at ZT8, *n* = 3 at ZT12, ZT16, and ZT20), and noradrenaline (**e**, *n* = 4 at ZT0, ZT4, and ZT8, and *n* = 3 at ZT12, ZT16, and ZT20) were measured. Data are shown as means ± SEM. The *p*-values were determined by two-way ANOVA with Holm-Sidak post hoc tests. *p < 0.05, **p < 0.01, ***p < 0.001.

Next, we subjected *Trpm6*^fl/fl^;*Six2-Cre* mice to blood pressure measurements using radiotelemetry. Systolic and diastolic blood pressure values were both significantly lower in *Trpm6*^fl/fl^;*Six2-Cre* mice than control mice (Fig. 2a and Supplementary Fig. 4). It is noteworthy that the usual circadian variation of blood pressure, with high values during the dark period and low values during the light period in mice, was not observed in *Trpm6*^fl/fl^;*Six2-Cre* mice. To examine whether the circadian rhythm proceeds normally, we used radiotelemetry to measure the locomotive activity of mice and confirmed a very clear circadian variation in *Trpm6*^fl/fl^;*Six2-Cre* mice (Fig. 2b). We also examined blood levels of vasopressin (AVP) and mRNA levels of two clock genes *Bmal1* and *Per2* in the brain and kidney; all are known to show typical circadian variation^27,28^. We analyzed at six different time points (at 0, 4, 8 zeitgeber times (ZT) for the light period and at 12, 16, 20 ZT for the dark period). Control mice show clear circadian patterns of their expression as previously reported^28^, and again, we found no significant abnormalities in *Trpm6*^fl/fl^;*Six2-Cre* mice (Fig. 2c and Supplementary Fig. 5). We also checked the expression level of *Trpm7*, which encodes an Mg^2+^-permeable channel closely related to TRPM6, in the kidney. The expression patterns were very similar in both *Trpm6*^+/+^;*Six2-Cre* and *Trpm6*^fl/fl^;*Six2-Cre* mice, and did not show clear circadian patterns (Supplementary Fig. 5). Collectively, we conclude that the circadian rhythm proceeds normally in *Trpm6*^fl/fl^;*Six2-Cre* mice. It has been considered that blood pressure elevation during the active period (the dark period for mice and the light period for humans) in both humans and mice occurs in response to an increase in renin secretion into the blood^2^. We measured renin activity in blood samples collected at the six different time points described above. A clear increase in blood renin activity was observed during the dark periods in samples from control mice, but no significant increase was observed in samples from those of *Trpm6*^fl/fl^;*Six2-Cre* mice (Fig. 2d). We also measured the blood level of noradrenaline, the release of which is known to be stimulated by the activation of renin–angiotensin system^29,30^. As expected, their circadian variation also disappeared in *Trpm6*^fl/fl^;*Six2-Cre* mice (Fig. 2e). Meanwhile, the protein level of blood angiotensinogen (AGT), another rate limiting molecule for activating renin–angiotensin system in mice^31^, was unaffected by *Trpm6* deficiency (Supplementary Fig. 6). The results suggest that the impaired circadian variation of renin is important for the loss of circadian variation of blood pressure by *Trpm6*-deficiency.

### Proximity of TRPM6-expressing cells to renin-secreting cells

**Fig. 3:**
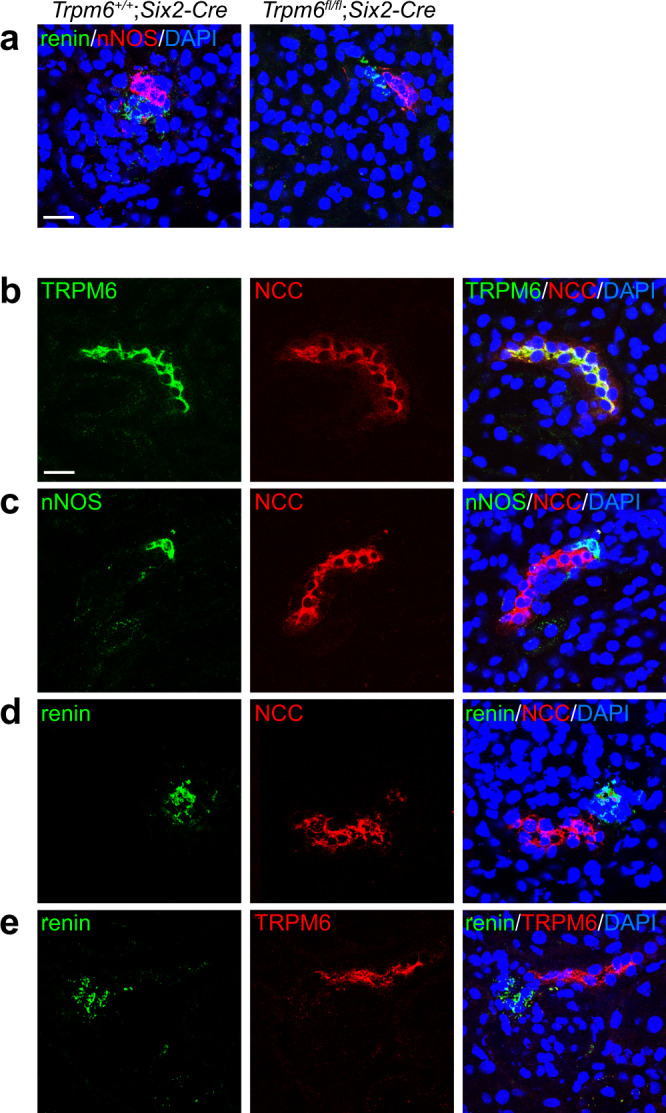
TRPM6-expressing cells are adjacent to renin-secreting cells. **a** Cryosections of 2-month-old *Trpm6*^+/+^;*Six2-Cre* and *Trpm6*^fl/fl^;*Six2-Cre* mouse kidneys were subjected to immunofluorescence staining with an anti-renin antibody (green), an anti-nNOS antibody (red), and DAPI (blue). Bar, 20 μm. A representative result from three independent experiments with similar results are shown. **b**–**e** Cryosections of 2-month-old *Trpm6*^+/+^;*Six2-Cre* mouse kidneys were subjected to immunofluorescence staining with the indicated antibodies and DAPI (blue). Merged images and monochrome images of each antibody staining are shown. Bar, 20 μm. A representative result from three independent experiments with similar results are shown.

Renin is expressed in and secreted from JG cells in the kidney^17,18^. JG cells are in direct contact with the macula densa, which is located adjacent to the DCT and which stimulates renin secretion from JG cells in response to the reduction of urinary Cl^−^ levels^18^. Immunofluorescence staining for renin and neuronal nitric oxide synthase (nNOS), a macula densa marker, showed no abnormalities in either the pattern or intensity of positive signals observed near the glomeruli in *Trpm6*^fl/fl^;*Six2-Cre* mice (Fig. 3a). Therefore, cells directly involved in renin secretion and its regulation do not seem to be markedly affected by *Trpm6* deficiency. As TRPM6 is co-expressed with NCC in the DCT (Supplementary Fig. 1c), we next examined the spatial relationship between TRPM6/NCC-expressing cells (DCT) and the macula densa or JG cells. Confocal images of sagittal DCT sections showed almost a complete overlap of TRPM6 and NCC signals throughout the DCT (Fig. 3b). We then stained for nNOS and NCC, and found that the areas positive for nNOS and NCC are connected, without any signal overlap (Fig. 3c). In addition, double staining for renin and TRPM6/NCC revealed that some TRPM6/NCC-expressing cells are in proximity to renin-expressing JG cells (Fig. 3d, e), suggesting that TRPM6/NCC-expressing cells, as well as the macula densa, may have a significant impact on renin secretion.

### Impaired renin secretion by *Trpm6* deficiency

**Fig. 4:**
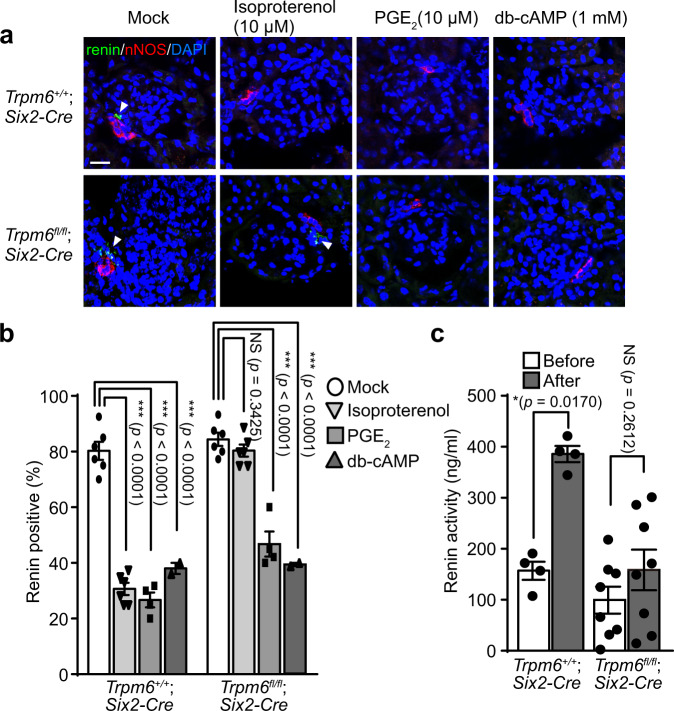
Impaired renin secretion upon adrenergic stimulation. **a** Slices of kidneys dissected from 2- to 3-month-old mice of the indicated genotypes were incubated with the indicated chemicals for 30 min and cryosections of these kidney slices were subjected to immunofluorescence staining with an anti-renin antibody (green), an anti-nNOS antibody (red), and DAPI (blue). Arrowheads indicate renin-positive cells. Bar, 20 μm. A representative result from three independent experiments with similar results are shown. **b** The percentages of glomeruli with adjacent renin-positive cells are shown as means ± SEM (in both genotype, *n* = 6 (mock), 6 (Isoproterenol), 4 (PGE_2_), and 2 (db-cAMP)). The *p*-values were determined by two-way ANOVA with Holm-Sidak post hoc tests. ***p < 0.001. **c** 2- to 3-month-old mice of the indicated genotypes were intraperitoneally administered with 10 mg/kg isoproterenol, and plasma collected the day before and 30 min after drug administration were subjected to renin activity measurement (*n* = 4 for *Trpm6*^+/+^; *Six2-Cre*, and *n* = 8 for *Trpm6*^fl/fl^; *Six2-Cre*). Data are shown as means ± SEM. The *p*-values were determined by two-way ANOVA with Holm-Sidak post hoc tests. *p < 0.05.

In general, the autonomic nervous system plays a crucial role in orchestrating the progression of the circadian rhythm. Renin secretion from JG cells during the active period is thought to be stimulated by noradrenalin secreted from renal sympathetic neurons^32^. To directly investigate the effect of such stimulation on renin secretion, we prepared kidney slices and stimulated them with isoproterenol, a β-adrenoreceptor (AR) agonist. Results showed a very clear decrease in renin-positive signal remaining in the slice after isoproterenol stimulation for 30 min (Fig. 4a). Quantitative analyses showed that the percentage of glomeruli surounded by renin-positive signal decreased from approximately 80% to 30% after isoproterenol stimulation (Fig. 4b), indicating the occurrence of massive renin secretion. However, we did not observe a significant effect of isoproterenol stimulation on renin signal in kidney slices from *Trpm6*^fl/fl^;*Six2-Cre* mice. To confirm the result, we also measured the blood renin activity after isoproterenol administration. The results show that significant elevation of blood renin activity was only observed in control mice (Fig. 4c), while the increase of heart rate was commonly observed in both strains (Supplementary Fig. 7). These results imply that renin-secreting JG cells cannot respond to stimulation from renal sympathetic neurons, consistent with the disappearance of the circadian increase in blood pressure and renin activity in *Trpm6*^fl/fl^;*Six2-Cre* mice.

JG cells can also be stimulated to secrete renin in response to prostaglandins, among which prostaglandin E_2_ (PGE_2_) is considered most significant since it is physiologically secreted by macula densa cells when urinary Cl^−^ levels decrease^17,18^. Moreover, it is also known that PGE_2_ treatment of kidney slices can stimulate renin secretion efficiently^33^. Stimulation of JG cells with PGE_2_ and isoproterenol commonly activates cAMP production, which ultimately causes renin secretion. Therefore, we also tested the effect of PGE_2_ and the membrane-permeable cAMP analog, dibutylyl-cAMP (db-cAMP). PGE_2_ and db-cAMP successfully stimulated renin secretion in kidney slices from *Trpm6*^fl/fl^;*Six2-Cre* mice and control mice (Fig. 4a, b). This indicates that *Trpm6* deficiency does not abrogate the general machinery involved in sensing external cues, relaying intracellular signals, and executing renin secretion, but it specifically affects the response to β-AR.

### Decreased β-AR expression in JG cells by *Trpm6* deficiency

**Fig. 5:**
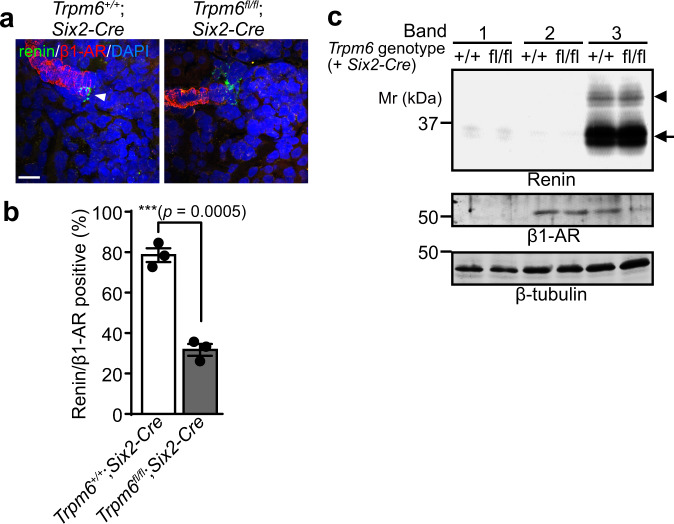
Decreased β1-AR expression in JG cell. **a** Cryosections of 2-month-old *Trpm6*^+/+^;*Six2-Cre* and *Trpm6*^fl/fl^;*Six2-Cre* mouse kidneys were subjected to immunofluorescence staining with an anti-renin antibody (green), an anti-β1-AR antibody (red), and DAPI (blue). Bar, 20 μm. A representative result from three independent experiments with similar results are shown. **b** The percentages of glomeruli with adjacent renin/β1-AR-double-positive cells are shown as means ± SEM (*n* = 3). The *p*-values were determined by Student’s two-tailed *t*-tests (unpaired). ***p < 0.001. **c** Kidneys dissected from 2-month-old *Trpm6*^+/+^;*Six2-Cre* and *Trpm6*^fl/fl^;*Six2-Cre* mice were treated with trypsin and collagenase, and dispersed cells were separated using Percoll density gradient centrifugation. Cells in each band were collected, and the cell lysates were subjected to immunoblotting with indicated antibodies. Arrowhead and arrow indicate precursor and processed mature form, respectively. A representative result from three independent experiments with similar results are shown.

Impaired β-AR receptor response (Fig. 4) in *Trpm6*^fl/fl^;*Six2-Cre* mice led us to investigate the status of the β-AR receptor expression, and thus we performed immunofluorescence of β1-AR, the major β-AR subtype expressed in JG cells^34^. As reported previously^34^, we found the band-like staining pattern in renal sections of both control and *Trpm6*^fl/fl^;*Six2-Cre* mice (Fig. 5a, b). It is known that β1-AR is strongly expressed in both blood vessel and JG cells^34^, and consistently, the β1-AR-positive cells at the end of the band-like staining in control mouse kidney were also positive for renin staining (shown with an arrowhead). In contrast, staining of *Trpm6*^fl/fl^;*Six2-Cre* mouse kidney showed a smaller number of β1-AR/renin double-positive cells, suggesting that β1-AR expression was decreased in JG cells. To corroborate this finding, we isolated JG cells by using Percoll density gradient centrifugation method according to the previous report^35^ (Fig. 5c). Immunoblotting analyses revealed that renin-positive JG cells are enriched in band 3 fraction, which is consistent with their report^35^, and β1-AR expression in this band 3 fraction was specifically much lower in *Trpm6*^fl/fl^;*Six2-Cre* mice. Taken together, we found that *Trpm6* deficiency decreases the expression level of β1-AR in JG cells, which is consistent with the impaired renin secretion.

### The effect of artificial intervention on circadian blood pressure variation

**Fig. 6:**
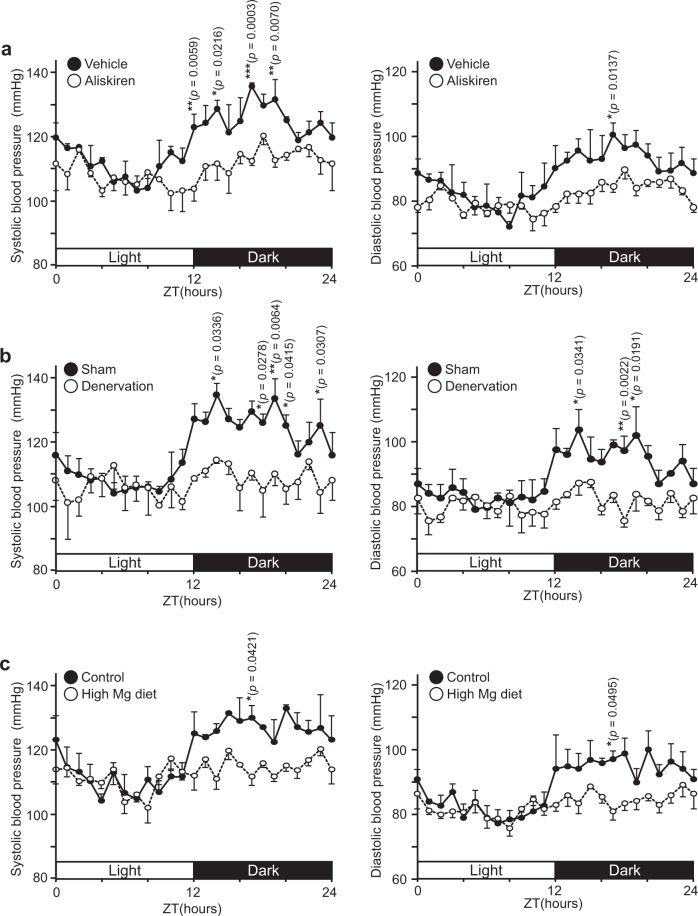
Effect of artificial intervention on circadian rhythms of blood pressure. 2- to 4-month-old WT mice were (**a**) administered with 25 mg/kg/day aliskiren via subcutaneously embedded osmotic minipump, **b** subjected to renal denervation, or **c** fed high-Mg diet, and blood pressure values were measured with radiotelemetry. Data are shown as means ± SEM for systolic (left) and diastolic (right) pressure values (*n* = 3 mice for each condition). The *p*-values were determined by two-way ANOVA with Holm-Sidak post hoc tests. *p < 0.05, **p < 0.01, ***p < 0.001.

In the final set of experiments, we tested the effect of various interventions on circadian blood pressure variation. To confirm the importance of renin, we administered a clinically available renin inhibitor aliskiren^36^ to wild-type mice (Fig. 6a). Like *Trpm6* ablation, aliskiren administration by surgically implanting an osmotic minipump significantly suppressed the circadian blood pressure variation. Next, we performed renal denervation operations, since the activation of β-AR on JG cells through the sympathetic nervous system is the major pathway for stimulating renin secretion in a circadian manner^32^ (Fig. 6b). Again, we found that the intervention suppressed the blood pressure variation. These results are in agreement with our model that defects in β-AR response and renin secretion are responsible for the loss of blood pressure variation in *Trpm6*^fl/fl^;*Six2-Cre* mice.

We also tested the effect of elevating dietary magnesium content. For this purpose, wild-type mice fed a high-Mg diet containing 0.6% (normal diet: 0.3% magnesium) for 1 month. As expected, those mice showed elevated blood Mg level (Supplementary Fig. 8a). We then checked the expression level of TRPM6 at DCT; it decreased drastically by feeding high-Mg diet (Supplementary Fig. 8b, c), and the extent of decreased TRPM6 expression was larger than renal *Cnnm2* ablation. When we measured the blood pressure in these mice, we found that mice fed a high-Mg diet showed significantly smaller blood pressure variation (Fig. 6c) as *Trpm6*-deficient mice, which is consistent with our notion that renal TRPM6 is important for circadian blood pressure variation.

## Discussion

In this study, we demonstrated the importance of TRPM6 in the regulation of blood pressure. In particular, we show almost complete disappearance of the circadian variation of blood pressure in kidney-specific *Trpm6*-deficient mice. We also found that secretion of renin, a key hormone orchestrating blood pressure, was severely abrogated in *Trpm6*-deficient mice, which is consistent with the observed blood pressure phenotype. Several reports have shown that TRPM7, another Mg^2+^-permeable channel closely similar to TRPM6, also affects blood pressure^37,38^. TRPM7 prevents the increase of blood pressure upon Angiotensin II administration by suppressing the response of vascular endothelial cells to Angiotensin II^37^. To the contrary, it was recently reported that TRPM7 also facilitates the elevation of blood pressure upon leptin administration by mediating Mg^2+^ influx into the carotid body cells^38^. Thus, the role of TRPM7 in blood pressure regulation is ambiguous. However, since the expression level of TRPM7 in the kidney was not affected by renal *Trpm6* ablation (Supplementary Fig. 5), it appears that TRPM7 is not directly involved in the phenotypes observed in this study.

As observed in *Cnnm2*-deficient mice, blood pressure values were reduced in *Trpm6*-deficient mice (Fig. 2)^14^. However, it should be noted that there was a clear difference between these mice. *Cnnm2* disruption generally reduced blood pressure, but circadian variation was still clearly observable. In contrast, this variation disappeared in *Trpm6*-deficient mice. Such a qualitative difference is also consistent with our observation that the circadian variation of renin was preserved in *Cnnm2*-deficient mice (Supplementary Fig. 9). It should be noted that both CNNM2 and TRPM6 are thought to be important for renal Mg^2+^ reabsorption by mediating the vectorial Mg^2+^ transport through the DCT, and indeed, mice deficient in either gene exhibited identical renal Mg^2+^-wasting phenotype (Fig. 1 and ref. ^39^). The qualitative difference of blood pressure phenotype between *Cnnm2*- and *Trpm6*-deficient mice suggests that Mg^2+^ transport in the DCT, the common function of the two molecules, is not directly involved in the circadian variation of blood pressure and renin secretion, suggesting that the CNNM2-independent role of TRPM6 underlies these intriguing phenotypes.

Several mechanisms work to stimulate renin secretion from JG cells to maintain blood pressure. Among them, the input from renal sympathetic neurons is considered to play the principal role in augmenting blood pressure during the active period of circadian variation^32^. These neurons secrete noradrenalin, which binds to and activates β-AR on JG cells to stimulate cAMP production, ultimately leading to renin secretion^17,32^. In our experiments using kidney slices obtained from *Trpm6*-deficient mice, stimulation with isoproterenol, a β-AR agonist, showed no effect on renin secretion, while stimulation with PGE_2_ or db-cAMP efficiently increased renin secretion (Fig. 4). PGE_2_ is secreted by the macula densa in response to low levels of urinary Cl^−^ and it activates EP2 and EP4 receptors on JG cells, which, like β-AR activation, ultimately stimulates cAMP production^40^. Therefore, these results imply that β-AR signaling is selectively abrogated in JG cells of *Trpm6*-deficient mice, without affecting the downstream cAMP-mediated signaling pathway. Consistently, immunofluorescence of mouse kidney sections and immunoblotting of isolated JG cells revealed that β-AR expression was significantly decreased in the JG cells of *Trpm6*-deficient mice (Fig. 5). Such reduced β-AR expression should make JG cells unable to secrete renin in response to sympathetic nerve stimulation during the active period, thereby eliminating circadian variation of blood renin level and blood pressure. It should be noted that there is a study using β1/β2-AR knockout mice, which showed decreased circadian blood pressure variation as *Trpm6*-deficient mice^41^. They ascribed it to the decreased locomotor activity, but it remains possible that reduced circadian variation of renin activity contributes to decreased circadian variation of blood pressure. Alternatively, the unusual background of their mice (mixed background of C57BL6/J, 129, and FVB; we used pure C57BL6/J background) might mask the effect of β-AR on blood pressure variation; genetic backgrounds are critical for circadian blood pressure variation^42^.

How the loss of TRPM6 in DCT cells leads to the decreased β-AR expression in JG cells has remained elusive. Experiments using other types of cells have shown that β-AR expression is regulated by various molecules known to mediate intercellular signaling: Addition of glucocorticoids to the culture medium of vas deferens-derived cell line DDT1 MF-2 increases β-AR expression^43^. Also, it was reported that nitric oxide (NO) suppressed β-AR degradation in cardiac cells through S-nitrosylation of GRK2^44^. Among these molecules, it is noteworthy that NO is secreted from renal macula densa cells, stimulating JG cells in a paracrine manner to increase renin secretion^45^. While the expression of nNOS, the NO producing enzyme in the kidney, is restricted to macula densa cells, they are in direct contact with DCT cells, the next segment of the renal tubule^16^. Thus, it is possible that *Trpm6*-deficiency in DCT cells leads to the decreased β-AR expression in JG cells by affecting the production or release of NO from the adjacent macula densa cells.

It is known that JG cells also communicate with other cells by forming gap junctions with surrounding cells, such as mesangial cells, and receive Ca^2+^ to suppress renin secretion^17^. Although JG cells and DCT cells do not directly contact with each other, they are still closely located (ref. ^46^ and Fig. 3) and it is possible that these cells use gap junctional communications through intervening cells, such as mesangial cells; all three cell types reportedly express Cx37^47,48^, a connexin isoform that oligomerizes and constitutes gap junctions. Further analysis may identify the molecule and the route responsible for the intercellular signaling between DCT and JG cells, which is important to unveil the overall mechanism of disrupted circadian blood pressure variation by *Trpm6*-deficiency.

In *Trpm6*-deficient mice, the circadian variation of blood levels of not just renin but also noradrenaline disappeared (Fig. 2e). Activation of the renin–angiotensin system stimulates the release of noradrenaline to the bloodstream^29,30^, and thus, it seems reasonable that the effect of *Trpm6* ablation on blood renin and noradrenaline levels was similar. Since noradrenaline also acts on the β-AR of JG cells to promote renin secretion, the two molecules are assumed to constitute a positive feedback loop, stimulating each other’s secretion. However, it should be noted that the results of isoproterenol administration experiments explicitly show the defect in renin secretion (Fig. 4), and thus it seems to be the primary cause of the loss of the circadian variation of both renin and noradrenalin.

Thus far, a variety of effective drugs have been developed and used to treat hypertensive patients. However, a significant number of patients show resistance to the conventional treatments that use a combination of major antihypertensive drugs of different functional mechanisms^49^. Surgical denervation of renal sympathetic neurons using endovascular catheters has been conducted on a trial basis to treat such resistant hypertension^50^. Its practical effectiveness at improving blood pressure control has been controversial^51,52^, but recently published studies have shown a significant reduction in blood pressure compared with sham controls^53,54^. There are also mixed results on the effect of renal denervation on circadian variation of blood pressure. Some reports showed that the extent of blood pressure decrease by renal denervation was larger during the active periods^53^, but others reported no change in circadian variation of blood pressure^55,56^. In the analysis using laboratory animals, our renal denervation operations against mice suppressed the circadian variation of blood pressure (Fig. 6). Similar operations were also performed against rats, but even control wild-type rats show almost no circadian blood pressure variation in the first place^57,58^, making it difficult to evaluate the effect of the operation. More studies are required to make clear the impact of renal denervation on circadian blood pressure variation, which would also help us to understand the more detailed mechanism of blood pressure dysregulation by *Trpm6*-deficiency.

## Methods

### Animals

*Cnnm2*^fl/fl^;*Six2-Cre*, *Cnnm2*^+/Δ^, *Cnnm4*^Δ/Δ^, and control mice were generated in our previous studies^14,20,59^. *Trpm6*-deficient mouse strains were generated from an ES cell clone carrying the recombinant allele of *Trpm6*, Trpm6^*−*^ (ID: EPD0741_2_G10, purchased from Knockout Mouse Project [KOMP]). Germline chimeras from this ES cell were bred with wild-type C57BL6/J mice to obtain *Trpm6*^+/*−*^ mice. *Trpm6*^+/*−*^ mice were then bred with *FlpO* mice^59^ to obtain mice with the *Trpm6*-floxed allele, Trpm6^fl^, and were subsequently crossed with *Six2-Cre* mice to obtain *Trpm6*^fl/fl^;*Six2-Cre* mice. Genotyping PCR was performed using the primers listed in Supplementary Table 1. Mice were maintained in individually ventilated cages in a specific pathogen-free, temperature controlled (20–26 °C) facility with a 12-h light/dark cycle. Age- and sex-matched male and female mice (2–4-month-old) were used in each independent experiment. All mouse experiments were conducted in accordance with the Guidelines of Proper Conduct of Animal Experiments (issued by the Science Council of Japan) after the receipt of approvals from the Animal Care and Use Committee of the Research Institute for Microbial Diseases, Osaka University, Japan.

### Expression constructs

Human *Trpm6* complementary DNA (cDNA) was purchased from Dharmacon (IMAGE: 100062461). The cDNA fragment was inserted into pCMV-Tag2B (Agilent) for expression in mammalian cells.

### Antibodies and chemicals

The anti-NCC guinea pig polyclonal antibody (1/500 dilution for immunofluorescence analyses) was kindly provided by Dr. Shinichi Uchida (Tokyo Medical and Dental University)^60^. Anti-CNNM2 (1/2,000 dilution for immunoblotting analyses and 1/200 for immunofluorescence analyses) and anti-CNNM4 (1/2,000 dilution for immunoblotting analyses) rabbit polyclonal antibodies were generated in our previous studies^14,20^. The anti-TRPM6 rabbit polyclonal antibody (1/300 dilution for immunofluorescence analyses) was generated by immunizing rabbits with the bacterially expressed His-tagged TRPM6 (1270–1453 a.a.) proteins and purified using the bacterially expressed GST-tagged TRPM6 recombinant proteins (1270–1497 a.a.). The following commercially available primary antibodies were used: anti-NCC antibody (AB3553; Millipore, 1/5,000 dilution for immunoblotting analyses), anti-phospho-NCC antibody (S995B; University of Dundee, 1/500 dilution for immunoblotting analyses), anti-TRPM6 antibody (AP8053C; Abgent, 1/5,000 dilution for immunoblotting analyses), anti-renin antibody (ISASRREN-GF; Innovative Research, 1/500 dilution for immunofluorescence analyses), anti-renin antibody (AF4277; R&D Systems, 1/1000 dilution for immunoblotting analyses), anti-nNOS antibody (160870; Cayman, 1/500 dilution for immunofluorescence analyses), anti-AGT antibody (28101; IBL, 1/100 dilution for immunoblotting analyses), anti-β1-AR antibody (PA1049; Invitrogen, 1/1000 dilution for immunoblotting analyses and 1/100 dilution for immunofluorescence analyses), anti-GFP antibody (A11122; Invitrogen, 1/3000 dilution for immunoblotting analyses), and anti-β-tubulin and anti-FLAG antibodies (T4026 and F1804, respectively; Sigma, both 1/5000 dilution for immunoblotting analyses). We used the following commercially available secondary antibodies: Alexa Fluor 488-conjugated anti-rabbit and anti-sheep antibodies and Alexa Fluor 568-conjugated anti-rabbit and anti-guinea pig antibodies (Life Technologies) for immunofluorescence analyses (all 1/2000 dilution), alkaline phosphatase-conjugated anti-rabbit, anti-goat, and anti-mouse antibodies (Promega, all 1/10,000 dilution), HRP-conjugated anti-sheep antibody (Pierce, 1/10,000 dilution), and Clean-Blot IP Detection Kit (for immunoprecipitated samples; Thermo Fisher, 1/200 dilution) for immunoblotting analyses.

We used the following commercially available chemicals: isoproterenol (I6504; Sigma), PGE_2_ (14010; Cayman), db-cAMP (023-16381; Wako), and aliskiren (A4534; LKT).

### Validation of anti-TRPM6 antibody

The specificity of the house made anti-TRPM6 antibody used for immunofluorescence analyses (Figs. 1b, 3b, and 3e and Supplementary Figs. 1c, 1e, and 8c) was validated by successive immunoprecipitation and immunoblotting analyses as follows (Supplementary Fig. 10)^61^. To reduce the background noise level due to the antibody itself, the anti-TRPM6 antibody was covalently linked to Protein A agarose beads (Thermo Fisher) by incubating with 20 mM dimethylpimelimidate (Sigma) for 30 min at room temperature. Then, the beads were mixed with lysates of cells or mouse organs. Lysates were prepared by solubilizing cells or tissues in lysis buffer (20 mM Tris-HCl [pH 7.5], 0.5% Triton X-100, 150 mM NaCl, 2 mM EDTA, supplemented with cOmplete Protease Inhibitor Cocktail [Roche]). The beads were rotated overnight at 4 °C, washed five times with lysis buffer, and the resultant immunoprecipitates were subjected to immunoblotting analyses. To reduce the background noise level due to the precipitated antibody, the Clean-Blot IP Detection Kit was used, which selectively reacts to native IgGs (primary antibodies) but not to denatured IgGs as secondary antibodies.

### Cell culture

MDCT cells^21^, kindly provided by Dr. Kouichi Tamura (Yokohama City University) and Dr. Peter A. Friedman (University of Pittsburgh), were cultured in Dulbecco’s modified Eagle’s medium (DMEM)/Ham’s F-12 Medium (1:1 mixture), supplemented with 5% fetal bovine serum (FBS) and antibiotics. HEK293 cells were routinely maintained in our laboratory and cultured in DMEM plus 10% FBS and antibiotics. Cell cultures under low-magnesium conditions were performed with magnesium/calcium-free DMEM (Thermo Fisher) supplemented with PBS-dialyzed FBS, 1.8 mM CaCl_2_, and the indicated concentrations of MgCl_2_. Expression plasmids and siRNAs were transfected into cells using LipofectAmine2000 (Invitrogen) or LipofectAmine RNAiMAX (Invitrogen), respectively.

### RNAi knockdown

Duplex siRNAs against mouse *Cnnm2* and *Cnnm4* were purchased from Sigma and Invitrogen, respectively. The target sequences were as follows: *Cnnm2*-siRNA, 5′-CCATTGTGCAGCGAGTGAA-3′ and *Cnnm4*-siRNA, 5′-GGTGGACGAG ACCACAACTCTT-3′. A non-targeting siRNA sequence was used as a control (negative control LO GC, Invitrogen).

### Elemental quantitation

Elemental quantification was done as follows^20^. Urine was collected by housing each mouse in metabolic cages (CLEA Japan) after 1 week acclimation. For serum samples, whole blood was collected from the abdominal aorta and incubated for 30 min at room temperature. Serum was then collected by centrifugation. Each element was quantified either by Xylidyl Blue-I (Mg, Wako) or ICP-ES (Mg, Ca, Na, and K; ICPS-8100; Shimadzu).

### Hormone quantitation

Whole blood was collected from the abdominal aorta in EDTA-containing tubes and plasma was isolated by centrifugation. Plasma renin activity was measured using a fluorescence renin assay kit (Abcam) and a microplate reader (SH-9000 controlled by SF61 software; both from Corona electric). Plasma AVP and noradrenaline levels were quantitated using Arg^8^-Vasopressin ELISA kit (Enzo Life Sciences) and Norepinephrine ELISA Kit (LDN), respectively, and the abovementioned microplate reader.

### Microarray analyses

Microarray analyses were performed with the following protocol^62^. Kidneys were dissected from 2-month-old *Cnnm2*^+/+^;*Six2-Cre* or *Cnnm2*^fl/fl^;*Six2-Cre* mice and total RNA was extracted using the RNeasy Extraction Kit (Qiagen). Dye-swapped experiments were performed by hybridizing complementary RNA (cRNA), labeled with either cyanine (Cy) -3 or Cy-5 (Perkin-Elmer), onto a Whole Mouse Genome Oligo Microarray 4 × 44 K ver.2 (G4846A; Agilent Technologies). Data were submitted to the NCBI Gene Expression Omnibus (GEO), with accession number, GSE73490. The genes (*p* < 0.1, two-tailed Student’s *t*-test, paired) with mean fold-changes > +2.0 or < −2.0 (“+” indicates upregulated and “−” indicates downregulated for each absolute value) in both experiments are listed in Table 1.

### PCR analyses

For cDNA synthesis^63^, RNA from mouse tissues or MDCT cells was prepared with RNAiso Plus (Takara), and cDNA was synthesized from 2 μg RNA with revertra ace (Toyobo). Semi-quantitative PCR analyses of mouse CNNMs^64^ were performed with a thermal cycler (T100; Bio-Rad) using Ex-taq (Takara). As a positive control, we also included samples using plasmid vectors containing the cDNA region of each mouse *Cnnm* as templates. Real-time qPCR experiments^62^ were performed with MiniOpticon (Bio-Rad) using the iQ SYBR green Supermix (Bio-Rad) as a reagent, and the data were obtained under the control of Opticon Monitor software (Bio-Rad). The quality of the final PCR product was confirmed by agarose gel electrophoresis. No obvious non-specifically amplified DNAs were observed. The primer sets used are listed in Supplementary Table 1.

### [Mg^2+^]_i_ measurement

For [Mg^2+^]_i_ measurement^20^, cells were loaded with 2 μM Mag-fura2-AM (Invitrogen) in serum-free DMEM for 45 min at 37 °C. After washing with serum-free DMEM, the cells were viewed using the IX81 microscope (Olympus) equipped with a CMOS camera (ORCA-Flash 4.0; Hamamatsu Photonics) and a mercury lamp (USH-1030L; Olympus). The fluorescence images were acquired under the control of metamorph software (Molecular Device) with excitation wavelength of 330 − 350 nm and 370 − 390 nm, and emission wavelength of 505 − 545 nm, respectively. [Mg^2+^]_i_ was determined from the following formula [Mg^2+^]_i_ = *K*_d_*Q* × (*R* − *R*_min_)/*R*_max_, where *R* is the ratio of the signal intensity with 330 − 350 nm excitation (*F*_1_) to that with 370 − 390 nm excitation (*F*_2_) (*R* = *F*_1_/*F*_2_), *R*_max_ is the maximum value of *R*, *R*_min_ is the minimum value of R, *Q* is the ratio of the signal intensity with 370 − 390 nm excitation when *R*_min_ was obtained to the signal intensity with 370 − 390 nm excitation when *R*_max_ was obtained (*F*_2min_/*F*_2max_), and we used 1.5 mM as the *K*_d_ value^20^. The value of *R*_min_, *R*_max_, *F*_2min_, and *F*_2max_ were obtained after each experiment. The cells were incubated in serum-free DMEM supplemented with 6 μM 4-Bromo-A23187 (Wako) and 10 mM EDTA, and the fluorescent images were acquired to obtain *R*_min_ and *F*_2min_ values. Subsequently, the media was replaced with serum-free DMEM supplemented with 6 μM 4-Bromo-A23187 and 50 mM MgCl_2_, and the fluorescent images were acquired to obtain *R*_max_ and *F*_2max_ values. For [Mg^2+^]_i_ measurement of cells cultured under various extracellular Mg^2+^ conditions, loading of Mag-fura2, washing, and data acquisition was performed by bathing the cells with magnesium/calcium-free DMEM (Thermo Fisher) supplemented with 1.8 mM CaCl_2_ and the indicated concentrations of MgCl_2_.

### Kidney slice preparation

By referring to several studies^65–67^, kidney slices were prepared as follows. Kidneys dissected from 2- to 3-month-old mice were immediately placed on ice-cold saline, and slices (0.4 mm thick) were cut using a vibratory tissue slicer (DTK-1000, Dosaka EM). Slices were preincubated in Krebs-Ringer bicarbonate solution (KRBS; 118.5 mM NaCl, 4.7 mM KCl, 2.5 mM CaCl_2_, 1.2 mM KH_2_PO_4_, 1.2 mM MgSO_4_, 25 mM NaHCO_3_, 10 mM Glucose, saturated with 95% O_2_/5% CO_2_ and adjusted to pH 7.4) at 37 °C for 15 min. After washing with KRBS, slices were incubated for a further 30 min at 37 °C in fresh KRBS containing chemical stimulants. Slices were then fixed with ice-cold 2% paraformaldehyde (PFA) in PBS for 2 h and subjected to immunofluorescence analyses.

### Immunofluorescence analyses of tissue sections

Whole kidneys or kidney slices from 2-month-old mice were embedded in OCT compound (Sakura Finetechnical), frozen in liquid nitrogen, and then cut into 20- or 30-µm sections using a cryostat (Leica). Sections were mounted on glass slides, air-dried, and fixed with ice-cold 2% PFA in PBS for 10 min. For subsequent immunostaining^62,64^, the sections were blocked twice in PBSMT (2% skim milk, 0.3% Triton X-100 in PBS) for 1 h at 4 °C. Then, the specimens were incubated with primary antibody diluted in PBSMT for overnight at 4 °C. After 7 washes with PBSMT, the specimens were incubated with appropriate fluorophore-conjugated secondary antibodies for overnight at 4 °C. Nuclei were also stained with 4’,6-diamidino-2-phenylindole (DAPI). After seven washes with PBSMT, the sections were observed with a confocal scanning laser microscope (FLUOVIEW FV1000; Olympus) under the control of FV10-ASW software (Olympus).

### Telemetric measurements of blood pressure, heart rate, and locomotor activity

Telemetric measurements of blood pressure were performed as follows^14^. Blood pressure transducers (TA11PA-C10, Data Sciences International) were surgically inserted into the left common carotid artery of 2- to 3-month-old mice under anesthesia (by intraperitoneal administration of 0.3 mg/kg medetomidine, 4 mg/kg midazolam, and 5 mg/kg butorphanol). After 3 weeks of recovery, systolic and diastolic blood pressure values were recorded with a 2-min scheduled sampling every 60 min by using Dataquest ART software (Data Sciences International). Records were obtained for at least five consecutive days, and the averages of measurements taken at the same time each day were determined for each mouse and used for further statistical analyses, except for Supplementary Fig. 4. Based on Nakamori et al.^68^, renal denervation was performed 2 weeks before inserting blood pressure transducers as follows. Kidneys were exposed by abdominal incision and connective tissue around the renal vessels was dissected. Then, the renal vessels were immersed in 95% ethanol for 2 min and thereafter, in PBS for 2 min. Sham operation was performed using the equivalent procedure without ethanol immersion. Aliskiren was administered 25 mg/kg/day by subcutaneously embedding an osmotic pump (1004, ALZET), and recording was started from the day after pump introduction. For magnesium supplementation, mice were fed a magnesium-enriched diet containing 0.6% magnesium (CLEA Japan) for 1 month before blood pressure measurement.

Heart rate was measured with the same radiotelemetry system, except that the recording was performed with a 30-s scheduled sampling every minute, and a single measurement was used for further analysis.

Locomotor activities were assessed using the same radiotelemetry system, by monitoring the changes in signal strength from the transducer due to the animal’s locomotion. In this case, transducers were surgically inserted into the subcutaneous space in the right flank. Recordings were performed with the same time intervals as described above.

### Isolation of JG cells

JG cells were isolated according to the method reported by Della Bruna and colleagues^35^, with several minor modifications. Kidneys from four mice were minced and incubated in buffer 1 (130 mM NaCl, 5 mM KC1, 2 mM CaCl_2_, 10 mM glucose, 20 mM sucrose, 10 mM Tris-HCl [pH 7.4]) supplemented with 0.25% trypsin and 0.1 % collagenase at 37 °C for 2 h. After filtration with a cell strainer (40 μm pore size, Falcon), cells were washed with buffer 1 and mixed with 20× volume of 30% isoosmotic Percoll solution. Centrifugation at 27,000 × *g* for 25 min yielded three bands, and each band was collected and used for further analyses.

### Statistical analyses

All statistical analyses were performed using Prism software (GraphPad) after data analyses with Microsoft Excel 11, Metamorph (Molecular Device), or Dataquest ART (Data science international). Circadian pattern was determined by cosinor analyses, which fits the data to the following formula *y*  = BaseLine + Amplitude × cos ([*x* – PhaseShift] × *π*/12). All statistical data are presented as means ± SEM and *p*-values were obtained by either Student’s two-tailed *t*-test, one- or two-way ANOVA with Holm-Sidak post hoc test, or chi-square test, as described in the figure legends.

### Reporting summary

Further information on research design is available in the Nature Research Reporting Summary linked to this article.

## Supplementary information

Supplementary Information

Peer Review File

Reporting Summary

## Data Availability

Microarray data were deposited to the NCBI Gene Expression Omnibus (GEO), with accession number GSE73490. The other source data supporting the findings of this study are publicly available in figshare [10.6084/m9.figshare.14544510]. Raw data are provided within source data file. Source data are provided with this paper.

## Source data

Source Data
