# Supplementary material for: Importance of the renal ion channel TRPM6 in the circadian secretion of renin to raise blood pressure
Source: Nat Commun. 2021 Jun 17;12:3683. doi: 10.1038/s41467-021-24063-2 (PMC8211686; doi:10.1038/s41467-021-24063-2)
Supplement: Supplementary file 1 — Supplementary Information [file 41467_2021_24063_MOESM1_ESM.pdf]

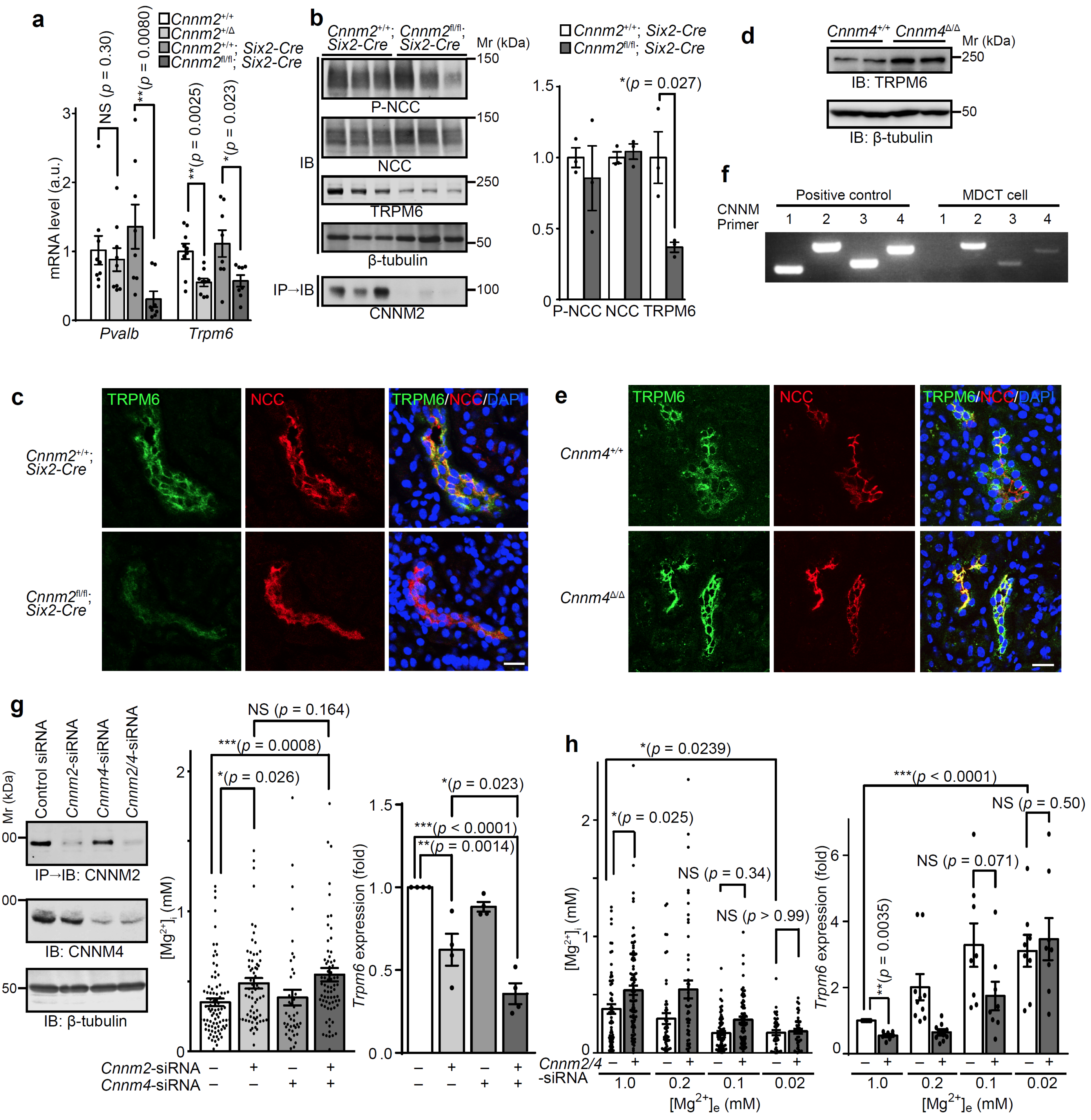
a
b
c
d
e
f
g
h
Cnnm2+/+
Cnnm2+/Δ
Cnnm2+/+; Six2-Cre
Cnnm2fl/fl; Six2-Cre
mRNA level (a.u.)
NS (p = 0.30)
**(p = 0.0080)
**(p = 0.0025)
*(p = 0.023)
Pvalb
Trpm6
Cnnm2+/+; Six2-Cre
Cnnm2fl/fl; Six2-Cre
Mr (kDa)
IB
P-NCC
NCC
TRPM6
β-tubulin
IP→IB
CNNM2
*(p = 0.027)
P-NCC NCC TRPM6
Cnnm4+/+
Cnnm4Δ/Δ
IB: TRPM6
IB: β-tubulin
CNNM Primer
Positive control
MDCT cell
TRPM6
NCC
TRPM6/NCC/DAPI
Control siRNA
Cnnm2-siRNA
Cnnm4-siRNA
Cnnm2/4-siRNA
IP→IB: CNNM2
IB: CNNM4
IB: β-tubulin
NS (p = 0.164)
***(p = 0.0008)
*(p = 0.026)
[Mg2+]i (mM)
Cnnm2-siRNA
Cnnm4-siRNA
Trpm6 expression (fold)
*(p = 0.023)
***(p < 0.0001)
**(p = 0.0014)
*(p = 0.0239)
*(p = 0.025)
NS (p = 0.34)
NS (p > 0.99)
Cnnm2/4 -siRNA
[Mg2+]e (mM)
***(p < 0.0001)
NS (p = 0.50)
NS (p = 0.071)
**(p = 0.0035)

Supplementary Figure 1 | Regulation of TRPM6 expression by CNNM and Mg^{2+}.

a, Kidneys were isolated from 2- to 3-month-old mice of the indicated genotypes and cDNA prepared from these kidneys was subjected to real-time PCR analyses for *Pvalb* and *Trpm6*. The relative expression levels of each gene are shown as means ± SEM (n = 9 for *Cnnm2*$^{+/+}$ and *Cnnm2*$^{+/\Delta}$ mice, and n = 8 for *Cnnm2*$^{+/+}$; *Six2-Cre* and *Cnnm2*$^{fl/fl}$; *Six2-Cre* mice). The *p* values were determined by Student's two-tailed t-tests (unpaired). $*p < 0.05$, $**p < 0.01$.

b, Kidney lysates were subjected to immunoprecipitation (IP) and immunoblotting (IB) analyses (for CNNM2) or direct IB analyses (for others) with the indicated antibodies. In the right panel, the signal intensities of phosphorylated-NCC (P-NCC), total NCC, and TRPM6 were determined by optical densitometry and their relative values are indicated as means ± SEM (n = 3). The *p* values were determined by Student's two-tailed t-tests (unpaired). $*p < 0.05$.

c, Cryosections of 2-month-old *Cnnm2*$^{+/+}$;*Six2-Cre* and *Cnnm2*$^{fl/fl}$;*Six2-Cre* mouse kidneys were subjected to immunofluorescence staining with an anti-TRPM6 antibody (green), anti-NCC antibody (red), and DAPI (blue). Bar, 20 μm. A representative result from 3 independent experiments with similar results are shown.

d, Kidney lysates from 4-month-old wild-type and *Cnnm4*-deficient mice were subjected to IB with an anti-TRPM6 antibody. A representative result from 2 independent experiments with similar results are shown.

e, Cryosections of kidneys from 4-month-old wild-type and *Cnnm4*-deficient mice were subjected to immunofluorescence staining with an anti-TRPM6 antibody (green), an anti-NCC antibody (red), and DAPI (blue). Bar, 20 μm. A representative result from 3 independent experiments with similar results are shown.

f, Total RNA was extracted from MDCT cells and semi-quantitative PCR analysis was performed for each CNNM isoform. The PCR results using each mouse CNNM cDNA as a template are also shown as positive controls.

g, MDCT cells transfected with the indicated siRNAs were loaded with Mag-fura2, and $[Mg^{2+}]_i$ was subsequently determined (center). Data are shown as means ± SEM (from left to right, n = 84, 65, 43, 70). The *p* values were determined by 1-way ANOVA with Holm-Sidak post hoc tests. $*p < 0.05$, $***p < 0.001$. *Trpm6* expression in each cell was determined by real-time PCR analyses (right). Data are shown as means ± SEM (n = 4). The *p* values were determined by 1-way ANOVA with Holm-Sidak post hoc tests. $**p < 0.01$, $***p < 0.001$. Representative results of IP and IB analyses for CNNM2 and direct IB analyses for CNNM4 are also shown (left).

h, MDCT cells were transfected with siRNAs for both *Cnnm2* and *Cnnm4* and were cultured in media with the indicated concentrations of Mg^{2+} for 16 h. $[Mg^{2+}]_i$ was subsequently determined (left), as in (**g**). Data are shown as means ± SEM (from left to right, n = 64, 97, 49, 49, 71, 68, 39, and 41). The *p* values were determined by 1-way ANOVA with Holm-Sidak post hoc tests. $*p < 0.05$, $**p < 0.01$, $***p < 0.001$. *Trpm6* expression was also determined (right), as in (**g**). Data are shown as means ± SEM (from left to right, n = 7, 7, 10, 10, 8, 8, 8, and 8). The *p* values were determined by 1-way ANOVA with Holm-Sidak post hoc tests. $**p < 0.01$, $***p < 0.001$.

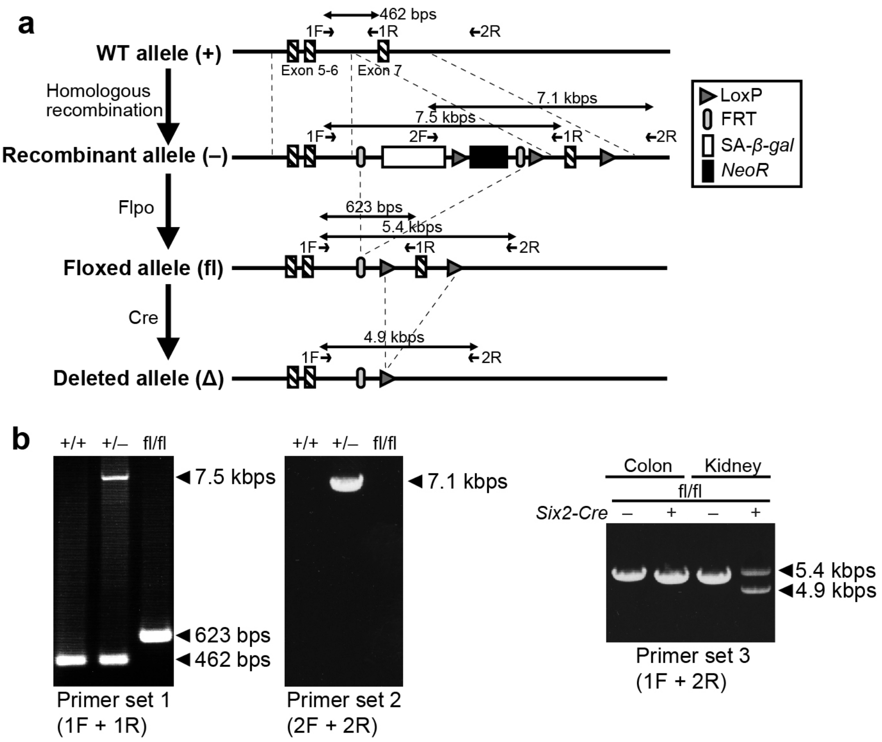

c Intercrosses of *Trpm6*$^{+/-}$ mice

n	+/+	+/–	–/–	*p* value
59	17	42	0	3.7 x 10^{-5}

Intercrosses of *Trpm6*$^{+/fl}$;*Six2-Cre* mice

n	Genotype (mice carrying *Six2-Cre*)			*p* value
	+/+	+/fl	fl/fl	
126	28	68	30	0.65

Supplementary Figure 2 | Generation of *Trpm6*$^{fl/fl}$;*Six2-Cre* mice.

a, Targeting strategy. LoxP, locus of crossing over of P1; FRT, Flp recombination target; SA, splice acceptor; *β-gal*, β-galactosidase gene; *NeoR*, neomycin resistance gene. The positions of PCR primers used for genotyping and the length of PCR products are indicated as arrows and bidirectional arrows, respectively.

b, Genomic DNA isolated from tails (left, center) or from the indicated tissues (right) of mice of the indicated *Trpm6* genotype were subjected to genotyping PCR with oligonucleotide primers schematically shown in (**a**).

c, Observed number of offspring (at P21) of each *Trpm6* genotype obtained by intercrosses of mice with the indicated genotypes. The *p* values were determined by Pearson's chi-square test.

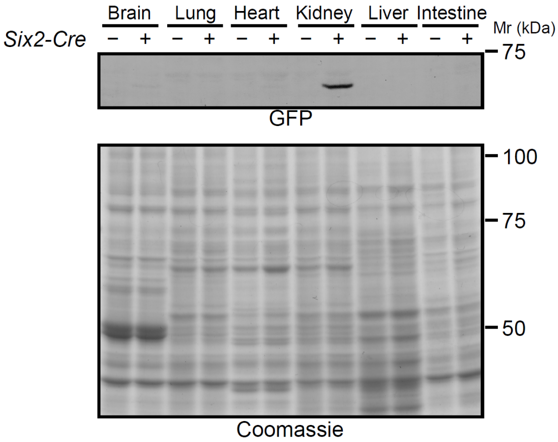

Supplementary Figure 3 | Kidney-specific expression of GFP-Cre fusion protein in *Six2-Cre* mouse.

Lysates obtained from various organs of E17.5 WT (−) or *Six2-Cre* (+) mouse were subjected to immunoblotting with an anti-GFP antibody to detect GFP-Cre fusion protein. A representative result from 3 independent experiments with similar results are shown.

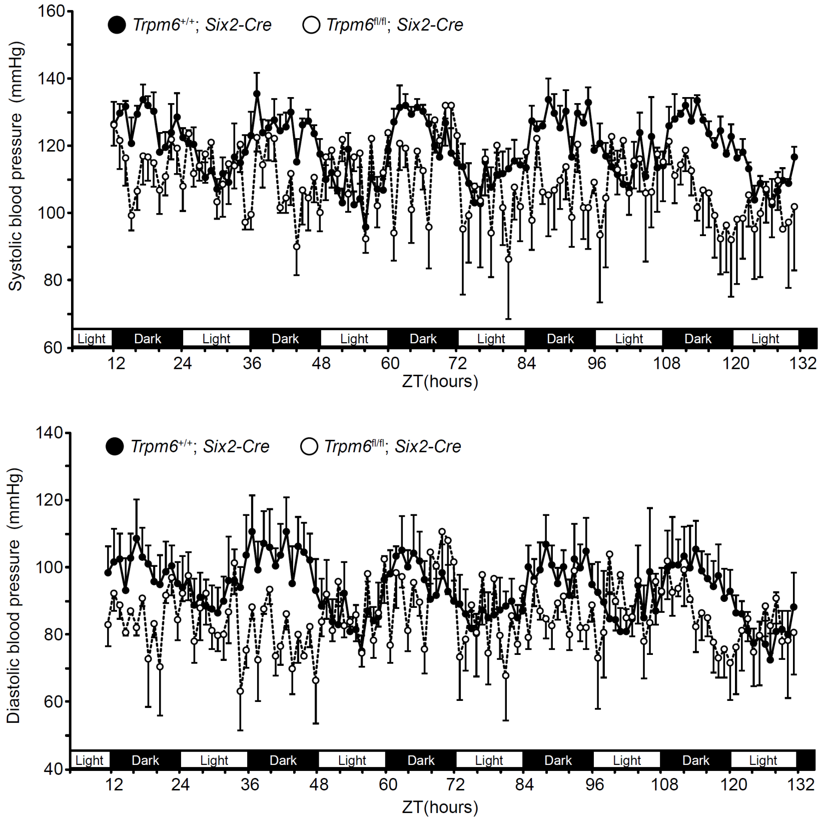

Supplementary Figure 4 | Time course variation of blood pressure over 5 days. Blood pressure values of 2- to 3-month-old mice of the indicated genotypes were measured with radiotelemetry. Data are shown as means ± SEM for systolic (top) and diastolic (bottom) pressure values over a period of 5 days. The averages of measurements taken at the same time each day are shown in **Figure 2a**.

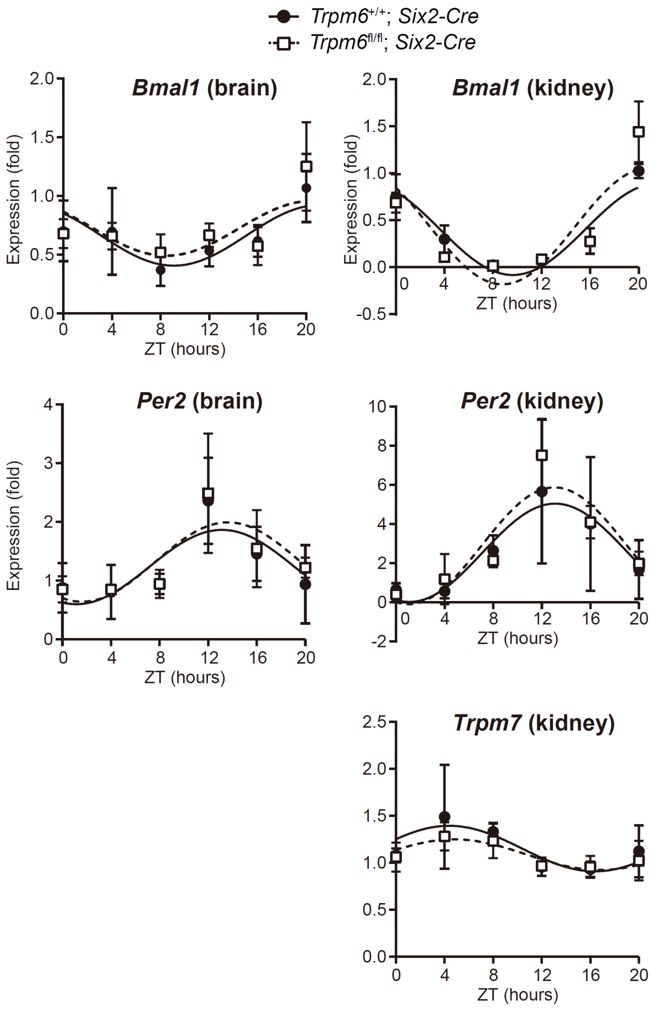

Supplementary Figure 5 | Expression and circadian variation of clock genes and *Trpm7*.

Total RNAs were collected from brain or kidney of 2- to 3-month-old mice of the indicated genotype (n = 3), and qPCR analyses for each gene were performed. Data are shown as means ± SEM, and lines represent the best-fitted curve determined by cosinor analysis.

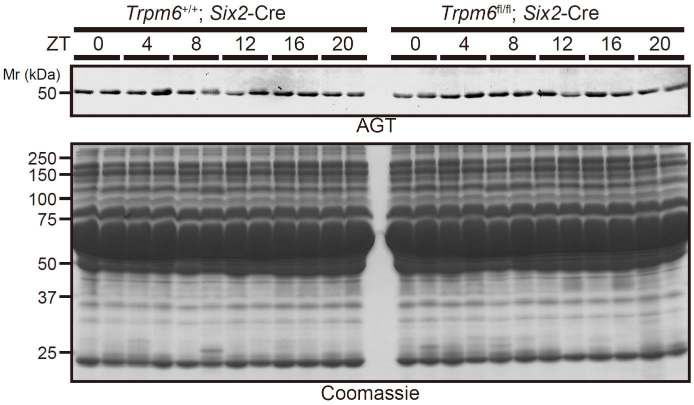

Supplementary Figure 6 | Expression analyses of plasma angiotensinogen (AGT). Plasma was collected from 2- to 3-month-old mice of the indicated genotype (n = 2), and subjected to immunoblotting analyses with an anti-AGT antibody.

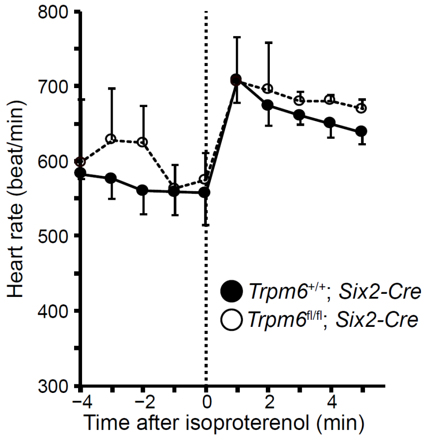

Supplementary Figure 7 | Heart rate elevation upon isoproterenol administration. 2-month-old mice of the indicated genotypes were intraperitoneally administered with 10 mg/kg isoproterenol (at 0 min), and heart rates were measured with radiotelemetry (n = 3). Data are shown as means ± SEM at each time point.

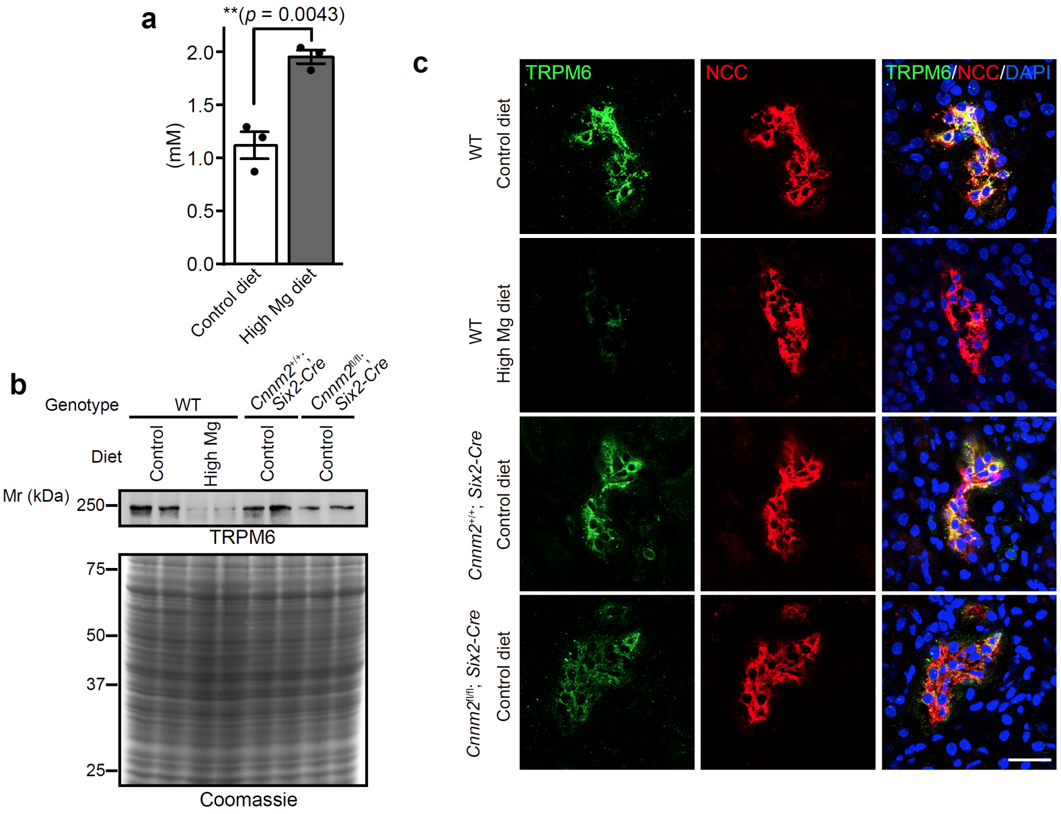

Supplementary Figure 8 | Effect of high Mg diet on renal TRPM6 expression.

a, Colorimetric quantitation of Mg in serum samples obtained from 2-month-old WT mice fed with indicated diet. Data are shown as means ± SEM (n = 3 mice). The *p* values were determined by Student's two-tailed t-tests (unpaired). $**p < 0.01$.

b, Kidney lysates from 2-month-old mice of indicated genotypes, which were fed with indicated diet, were subjected to immunoblotting analyses with an anti-TRPM6 antibody. A representative result from 3 independent experiments with similar results are shown.

c, Cryosections of kidneys from 2-month-old mice of indicated genotypes, which were fed with indicated diet, were subjected to immunofluorescence staining with an anti-TRPM6 antibody (green), an anti-NCC antibody (red), and DAPI (blue). Bar, 20 µm. A representative result from 3 independent experiments with similar results are shown.

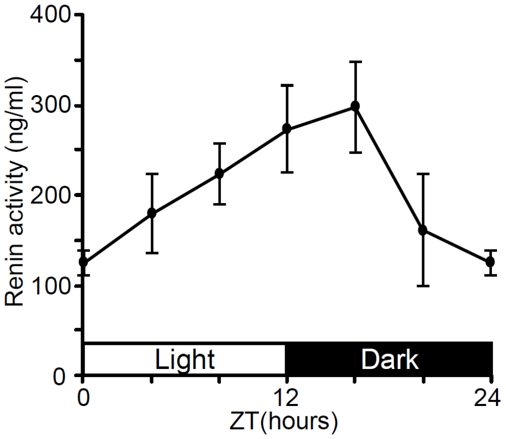

Supplementary Figure 9 | Circadian variation of blood renin activity in *Cnnm2*$^{fl/fl}$;*Six2-Cre* mice.

Whole blood was collected from 2- to 4-month-old *Cnnm2*$^{fl/fl}$;*Six2-Cre* mice at indicated time periods, and plasma renin activity (n = 3) were measured. Data are shown as means ± SEM.

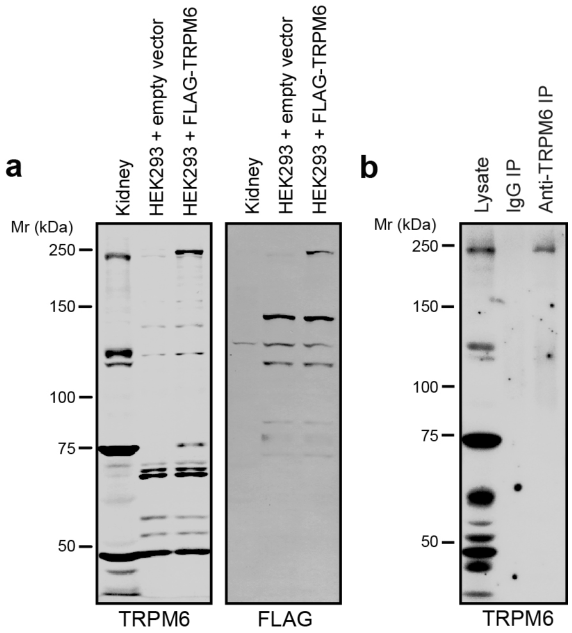

Supplementary Figure 10 | Validation of anti-TRPM6 antibody used for immunofluorescence analyses.

a, Lysates of kidney or HEK293 cells transfected with the indicated constructs were subjected to immunoblotting with anti-TRPM6 and anti-FLAG antibodies. A representative result from 2 independent experiments with similar results are shown.

b, Kidney lysates were subjected to anti-TRPM6 immunoprecipitation (IP), and immunoprecipitates were analyzed by anti-TRPM6 immunoblotting. A representative result from 2 independent experiments with similar results are shown.

Primer	Sequence
Trpm6 genotyping 1F	5′-GGACAGTTTCTCCTCTCTCTGCTCC-3′
Trpm6 genotyping 1R	5′-ACAATACCCACACATATCCTGCCCC-3′
Trpm6 genotyping 2F	5′-CACACCTCCCCCTGAACCTGAAAC-3′
Trpm6 genotyping 2R	5′-CTTGAGTACTCATTACAAGCATGAACTG-3′
Cnnm1 Fwd	5′-GCCGAGATCTGTCCGTACTC-3′
Cnnm1 Rev	5′-GCAGCTTTTCCCTCGTGTAG-3′
Cnnm2 Fwd	5′-GGAGAACGTTCCAACATCGT-3′
Cnnm2 Rev	5′-GCTGTCCGTCTGCTTAAAGG-3′
Cnnm3 Fwd	5′-CCGGCACTGTCCTAGACTTC-3′
Cnnm3 Rev	5′-GCGTCCAGTTTGGTATCGTT-3′
Cnnm4 Fwd	5′-GCTTCTACAACCACCCGGTA-3′
Cnnm4 Rev	5′-AGCAGCCAGAAGAAGCTGAG-3′
Pvalb Fwd	5′-CGCTGAGGACATCAAGAAGG-3′
Pvalb Rev	5′-CCGGGTTCTTTTTCTTCAGG-3′
Trpm6 Fwd	5′-AAAGCCATGCGAGTTATCAGC-3′
Trpm6 Rev	5′-CTTCACAATGAAAACCTGCCC-3′
Bmal1 Fwd	5′-GCAGTGCCACTGACTACCAAGA-3′
Bmal1 Rev	5′-TCCTGGACATTGCATTGCAT-3′
Per2 Fwd	5′-GGCTTCACCATGCCTGTTGT-3′
Per2 Rev	5′-GGAGTTATTTCGGAGGCAAGTGT-3′
Trpm7 Fwd	5′-TTTGGTGTTCCCAGAAAAGC-3′
Trpm7 Rev	5′-ACCAAGTTCCAGGACCACAG-3′
Gapdh Fwd	5′-TAACATCAAATGGGGTGAGG-3′
Gapdh Rev	5′-GGTTCACACCCATCACAAAC-3′

Supplementary Table 1 | List of primers used in this study.
