# Supplementary material for: Importance of the renal ion channel TRPM6 in the circadian secretion of renin to raise blood pressure
Source: Nat Commun. 2021 Jun 17;12:3683. doi: 10.1038/s41467-021-24063-2 (PMC8211686; doi:10.1038/s41467-021-24063-2)
Supplement: Supplementary file 3 — Reporting Summary [file 41467_2021_24063_MOESM3_ESM.pdf]

nature research

Corresponding author(s): Hiroaki Miki

Last updated by author(s): May 7, 2021

# Reporting Summary

Nature Research wishes to improve the reproducibility of the work that we publish. This form provides structure for consistency and transparency in reporting. For further information on Nature Research policies, see our Editorial Policies and the Editorial Policy Checklist.

## Statistics

For all statistical analyses, confirm that the following items are present in the figure legend, table legend, main text, or Methods section.

| n/a | Confirmed | |
|---|---|---|
| ☐ | ☒ | The exact sample size (*n*) for each experimental group/condition, given as a discrete number and unit of measurement |
| ☐ | ☒ | A statement on whether measurements were taken from distinct samples or whether the same sample was measured repeatedly |
| ☐ | ☒ | The statistical test(s) used AND whether they are one- or two-sided<br>*Only common tests should be described solely by name; describe more complex techniques in the Methods section.* |
| ☒ | ☐ | A description of all covariates tested |
| ☐ | ☒ | A description of any assumptions or corrections, such as tests of normality and adjustment for multiple comparisons |
| ☐ | ☒ | A full description of the statistical parameters including central tendency (e.g. means) or other basic estimates (e.g. regression coefficient) AND variation (e.g. standard deviation) or associated estimates of uncertainty (e.g. confidence intervals) |
| ☐ | ☒ | For null hypothesis testing, the test statistic (e.g. *F*, *t*, *r*) with confidence intervals, effect sizes, degrees of freedom and *P* value noted<br>*Give P values as exact values whenever suitable.* |
| ☒ | ☐ | For Bayesian analysis, information on the choice of priors and Markov chain Monte Carlo settings |
| ☒ | ☐ | For hierarchical and complex designs, identification of the appropriate level for tests and full reporting of outcomes |
| ☒ | ☐ | Estimates of effect sizes (e.g. Cohen's *d*, Pearson's *r*), indicating how they were calculated |

*Our web collection on statistics for biologists contains articles on many of the points above.*

## Software and code

Policy information about availability of computer code

| | |
|---|---|
| Data collection | Metamorph (Molecular Device v. 7.8.4.0), FV10-ASW (Olympus v. 04.02.03.06), Opticon Monitor (Bio-Rad v. 3.1.32), ICPS-8100 (Shimadzu v. 1.08), SF61 (Corona electric v. 4.2.4), Dataquest ART (Data science international v. 4.35) |
| Data analysis | Prism (GraphPad v. 6.07), Metamorph (Molecular Device v. 7.8.4.0), Dataquest ART (Data science international v. 4.35), Microsoft Excel 2011 (v. 16.0.4266.1001). |

For manuscripts utilizing custom algorithms or software that are central to the research but not yet described in published literature, software must be made available to editors and reviewers. We strongly encourage code deposition in a community repository (e.g. GitHub). See the Nature Research guidelines for submitting code & software for further information.

## Data

Policy information about availability of data

All manuscripts must include a data availability statement. This statement should provide the following information, where applicable:

- Accession codes, unique identifiers, or web links for publicly available datasets
- A list of figures that have associated raw data
- A description of any restrictions on data availability

Microarray data were deposited to the NCBI Gene Expression Omnibus (GEO), with accession number GSE73490 [https://www.ncbi.nlm.nih.gov/geo/query/acc.cgi?acc=GSE73490]. The other source data supporting the findings of this study are publicly available in figshare [https://dx.doi.org/10.6084/m9.figshare.14544510].

# Field-specific reporting

Please select the one below that is the best fit for your research. If you are not sure, read the appropriate sections before making your selection.

☒ Life sciences ☐ Behavioural & social sciences ☐ Ecological, evolutionary & environmental sciences

For a reference copy of the document with all sections, see nature.com/documents/nr-reporting-summary-flat.pdf

# Life sciences study design

All studies must disclose on these points even when the disclosure is negative.

| | |
|---|---|
| Sample size | No statistical methods were used to predetermine the sample size. Sample sizes were determined based on previous studies in the related research field (e.g. PLoS Genet. 9, e1003983 (2013), J. Clin. Invest. 124, 5398–5410 (2014), J. Hypertens. 35, 585–592 (2017), Nat. Commun. 3, 1285 (2012)). |
| Data exclusions | We did not exclude any data points. |
| Replication | The experimental findings in all figures were reproduced successfully. The number of the independent experiments performed are described in the figure legends. |
| Randomization | We did not randomize our experiments, but independent mice or cell cultures were used for each independent repeat and/or done on different days. |
| Blinding | In most experiments, data acquisition and analyses were done by the same person who performed the experiments, and thus, our study was not done in a blinded manner. To minimize the bias, the samples were treated as equally and data collection and/or analysis were mainly performed by computer based method. |

# Reporting for specific materials, systems and methods

We require information from authors about some types of materials, experimental systems and methods used in many studies. Here, indicate whether each material, system or method listed is relevant to your study. If you are not sure if a list item applies to your research, read the appropriate section before selecting a response.

## Materials & experimental systems

| n/a | Involved in the study | |
|---|---|---|
| ☐ | ☒ | Antibodies |
| ☐ | ☒ | Eukaryotic cell lines |
| ☒ | ☐ | Palaeontology and archaeology |
| ☐ | ☒ | Animals and other organisms |
| ☒ | ☐ | Human research participants |
| ☒ | ☐ | Clinical data |
| ☒ | ☐ | Dual use research of concern |

## Methods

| n/a | Involved in the study | |
|---|---|---|
| ☒ | ☐ | ChIP-seq |
| ☒ | ☐ | Flow cytometry |
| ☒ | ☐ | MRI-based neuroimaging |

## Antibodies

Antibodies used

Anti-NCC guinea pig polyclonal antibody: provided by Dr. Shinichi Uchida (Tokyo Medical and Dental University)
Anti-CNNM2 rabbit polyclonal antibody (generated in our previous study; J. Hypertens. 35, 585–592 (2017))
Anti-CNNM4 rabbit polyclonal antibodies (generated in our previous study; PLoS Genet. 9, e1003983 (2013)).
Anti-TRPM6 rabbit polyclonal antibody (generated in this study)
Anti-NCC rabbit polyclonal antibody (AB3553; Millipore)
Anti-phospho-NCC sheep polyclonal antibody (S995B; University of Dundee)
Anti-TRPM6 rabbit polyclonal antibody (AP8053C; Abgent)
Anti-renin sheep polyclonal antibody (ISASRREN-GF; Innovative Research)
Anti-renin goat polyclonal antibody (AF4277; R&D Systems)
Anti-nNOS rabbit polyclonal antibody (160870; Cayman)
Anti-AGT rabbit polyclonal antibody (28101; IBL)
Anti-β1-AR rabbit polyclonal antibody (PA1049; Invitrogen)
Anti-GFP rabbit polyclonal antibody (A11122; Invitrogen)
Anti-β-tubulin mouse monoclonal antibody (clone TUB 2.1, T4026; Sigma)
Anti-FLAG mouse monoclonal antibody (clone M2, F1804; Sigma)
Alexa Fluor 488-conjugated anti-rabbit antibody (A-11034; Life Technologies)
Alexa Fluor 488-conjugated anti-sheep antibody (A-11015; Life Technologies)
Alexa Fluor 568-conjugated anti-rabbit antibody (A-11036; Life Technologies)
Alexa Fluor 568-conjugated anti-guinea pig antibody (A-11075; Life Technologies)

| | |
|---|---|
| | Alkaline phosphatase-conjugated anti-rabbit antibody (S3731; Promega)<br>Alkaline phosphatase-conjugated anti-goat antibody (V1151; Promega)<br>Alkaline phosphatase-conjugated anti-mouse antibody (S3721; Promega)<br>HRP-conjugated anti-sheep antibody (SA1-74002; Pierce)<br>Clean-Blot IP Detection Kit (21232; Thermo Fisher) |

Validation

As for our house made antibodies, the validation data are provided in this paper (for anti-TRPM6, suplemantary figure 10) or in our previous papers (PLoS Genet. 9, e1003983 (2013) for anti-CNNM4 and J. Hypertens. 35, 585–592 (2017) for anti-CNNM2 antibodies, respectively). Validation data for anti-NCC (kindly provided by Dr. S. Uchida) are shown in the paper from their group (Histochem. Cell Biol. 136, 25–35 (2011)). The links of the validation profiles for each commercially available antibody in conjunction with citations from other works using them are shown below.

Anti-NCC rabbit polyclonal antibody: https://www.merckmillipore.com/JP/ja/product/Anti-Thiazide-Sensitive-NaCl-Cotransporter-Antibody,MM_NF-AB3553
Anti-phospho-NCC sheep polyclonal antibody: https://mrcppureagents.dundee.ac.uk/reagents-view-antibodies/588276
Anti-TRPM6 antibody: https://search.cosmobio.co.jp/view/p_view.asp?PrimaryKeyValue=7018743&ServerKey=&selPrice=1&Usq=@s@MAKER:ABGHIN:AP8053CSIZE:@e@
Anti-renin sheep polyclonal antibody: https://www.innov-research.com/products/sheep-anti-mouse-rat-prorenin-renin-polyclonal-affinity-purified-immobilized
Anti-renin antibody: https://www.rndsystems.com/products/mouse-renin-1-antibody_af4277
Anti-nNOS antibody: https://www.caymanchem.com/product/160870/nnos-polyclonal-antibody
Anti-AGT antibody: https://www.ibl-japan.co.jp/search/product/detail/id=3826
Anti-β1-AR antibody: https://www.thermofisher.com/antibody/product/beta-1-Adrenergic-Receptor-Antibody-Polyclonal/PA1-049
Anti-GFP rabbit polyclonal antibody: https://www.thermofisher.com/antibody/product/GFP-Antibody-Polyclonal/A-11122
Anti-β-tubulin mouse monoclonal antibody: https://www.sigmaaldrich.com/catalog/product/Sigma/T4026?lang=ja&region=JP&gclid=Cj0KCQjwvr6EBhDOARIsAPpqUPH_2MT8yyydRyPXRFbTM3vpF31WpZ_HPG6nXSv5Ab4MiqljT0awE2IaAgopEALw_wcB
Anti-FLAG mouse monoclonal antibody: https://www.sigmaaldrich.com/catalog/product/sigma/f1804?lang=ja&region=JP
Alexa Fluor 488-conjugated anti-rabbit antibody: https://www.thermofisher.com/antibody/product/Goat-anti-Rabbit-IgG-H-L-Highly-Cross-Adsorbed-Secondary-Antibody-Polyclonal/A-11034
Alexa Fluor 488-conjugated anti-sheep antibody: https://www.thermofisher.com/antibody/product/Donkey-anti-Sheep-IgG-H-L-Cross-Adsorbed-Secondary-Antibody-Polyclonal/A-11015
Alexa Fluor 568-conjugated anti-rabbit antibody: https://www.thermofisher.com/antibody/product/Goat-anti-Rabbit-IgG-H-L-Highly-Cross-Adsorbed-Secondary-Antibody-Polyclonal/A-11036
Alexa Fluor 568-conjugated anti-guinea pig antibody: https://www.thermofisher.com/antibody/product/Goat-anti-Guinea-Pig-IgG-H-L-Highly-Cross-Adsorbed-Secondary-Antibody-Polyclonal/A-11075
Alkaline phosphatase-conjugated anti-rabbit antibody: https://www.promega.com/products/protein-detection/primary-and-secondary-antibodies/anti-rabbit-igg-fc-ap-conjugate/?catnum=s3731,S3731&cs=y&catNum=S3731
Alkaline phosphatase-conjugated anti-goat antibody: https://www.promega.com/products/protein-detection/primary-and-secondary-antibodies/donkey-anti-goat-igg-ap-conjugate/?catNum=V1151&cs=y
Alkaline phosphatase-conjugated anti-mouse antibody: https://www.promega.com/products/protein-detection/primary-and-secondary-antibodies/anti-mouse-igg-h-and-l-ap-conjugate/?catnum=s3721&catNum=S3721
HRP-conjugated anti-sheep antibody: https://www.fishersci.se/shop/products/donkey-anti-sheep-igg-h-l-chains-secondary-antibody-hrp-conjugate/11585170
Clean-Blot IP Detection Kit: https://www.thermofisher.com/order/catalog/product/21232#/21232

## Eukaryotic cell lines

Policy information about cell lines

| | |
|---|---|
| Cell line source(s) | MDCT cells were kindly provided by Dr. Kouichi Tamura (Yokohama City University) and Dr. Peter A. Friedman (University of Pittsburgh). HEK293 cells were originally obtained from ATCC and routinely maintained in our laboratory. |
| Authentication | Cell lines were not independently authenticated. |
| Mycoplasma contamination | Cell lines are not mycoplasma contaminated. |
| Commonly misidentified lines (See ICLAC register) | No commonly misidentified lines were used in this study. |

## Animals and other organisms

Policy information about studies involving animals; ARRIVE guidelines recommended for reporting animal research

| | |
|---|---|
| Laboratory animals | Wild type, CNNM2-deficient, and TRPM6-deficient mice were on the C57BL/6J background. Mice between 2-3 months old were analyzed unless otherwise mentioned. Both male and female mice were used in this study. All mice were maintained in individually ventilated cages in a specific pathogen-free, temperature controlled (20–26°C) facility with a 12-h light/dark cycle. |
| Wild animals | Wild animals were not used in this study. |
| Field-collected samples | Field-collected samples were not used in this study. |
| Ethics oversight | All mouse experiments were conducted in accordance with the Guidelines of Proper Conduct of Animal Experiments (issued by the |

| | |
|---|---|
| Ethics oversight | Science Council of Japan) after the receipt of approvals from the Animal Care and Use Committee of the Research Institute for Microbial Diseases, Osaka University, Japan. |

Note that full information on the approval of the study protocol must also be provided in the manuscript.
