# Supplementary material for: Importance of the renal ion channel TRPM6 in the circadian secretion of renin to raise blood pressure
Source: Nat Commun. 2021 Jun 17;12:3683. doi: 10.1038/s41467-021-24063-2 (PMC8211686; doi:10.1038/s41467-021-24063-2)
Supplement: Supplementary file 2 — Peer Review File [file 41467_2021_24063_MOESM2_ESM.pdf]

Reviewers' comments:

Reviewer #1 (Remarks to the Author):

The manuscript by Funato et al. reports that conditional deletion of trpm6 in the kidney is associated with hypotension and the absence of circadian blood pressure cycling. From the results of further experiments the authors speculate, that the absence of diurnal blood presure changes, in particular, the increase of blood blood pressure during the dark phase result from an impaired beta-adrenergic stimulability of renin secretion from kidneys with conditional trpm6 deletion.

Although I wish to appreciate the successful generation of a conditional renal trpm6 knockout, I have major concerns regarding the other findings and speculations.

-demonstration of one diurnal blood pressure cycle is not sufficient. Recordings for 5-7 subsequent days should be shown to allow an impression about the constancy of differences between the two genotypes
- the speculation that the almost complete absence of circadian blood pressure changes could be the result of impaired renin secretion appears somewhat far fetched. I wonder if inhibition of the RAS by ACE-inhibitors or sartans really abolishes blood pressure cycles. Therefore the authors should support their speculations by demonstrating the effects of RAS-inhibitors on blood pressure cycles.
- the method used to measure renin secretion from the kidney (fig.6) is really not state of the art. It is strongly recommended to determine renin secretion either from isolated perfused kidney or in vivo after injection or infusion of catecholamines. In this context the concentration of the drugs used must be given.
-if it would turn out that beta-adrenergic signaling in renin secreting cells of trpm6 ko kidneys is impaired it should be examined if this is a renin cell specific effect or holds for all beta-1 adrenergic receptors (for example heart rate after catecholamine administration
- it should be checked, if the possible effects of trpm6 deletion on blood pressure and on beta-adrenergic signaling can be compensated by magnesium supplementation

Reviewer #2 (Remarks to the Author):

This study by Funato et al investigated the role of TRPM6 in blood pressure regulation by studying kidney-specific Trpm6-deficient mice. Results demonstrate that Trpm6-deficient mice have reduced blood pressure and a blunted circadian rhythm. This was associated with reduced agonist-stimulated renin secretion in kidney sections. Based on these findings the authors conclude that TRPM6 plays a critical role in diurnal blood pressure regulation and this involves renin. This is an interesting study that combines mouse models and in vitro studies. However there are a number of aspects that warrant further consideration.

Specific comments
1. This study builds on previous findings by the group where it was shown that in Cnnm2-deficient mice, renal Trpm6 expression is reduced with associated decrease in blood pressure but normal circadian rhythm. Here it is shown the Trpm6-deficient mice have reduced blood pressure with loss of circadian rhythm. In both conditions Trpm6 is reduced with decreased $Mg2+$, hence the altered circadian rhythm may be due to factors other than renal Trpm6 downregulation and hypomagnesemia. Please discuss this in the context of the present studies.

2. The studies relating to Cnnm2 (figures 1 and 2) do not contribute significantly to the overall goals of the present study. Also, it is unclear exactly how Cnnm2 and Trpm6 interact in the kidney and whether their interaction is simply secondary to changes in intracellular Mg. Please expand.

3. A major finding in this study is the lack of circadian rhythm in the Trpm6-deficient mice. However it should be noted that the blood pressures in the wild type mice are high, reaching levels levels as high as 160/120 mmHg during the dark periods (figure 4a, 4b). These pressures, measured by telemetry are pathological and by definition, the mice would be considered to be hypertensive. Moreover, even at baseline (time 0), blood pressures seem high (normal blood

pressure in mice is about 110/80 mmHg, and may be lower with telemetry readings). Hence, there are concerns regarding the Trpm6+/+ control mice.

4. The studies related to kidney renin production in basal and stimulated states are superficial and are based on qualitative (at most semi-quantitative) methods using immunofluorescence. This is sub-optimal because major conclusions are made in this study relating to renin.

5. As stated above, all the renin studies are qualitative based on immunofluorescence approaches (figs 5, 6). More specific assays for renin measurement in tissues/cells need to be included. This is critical considering the conclusions of the study.

6. It would be useful to know what happens to renin production in kidneys from Cnnm2 mice, in which Trpm6 is downregulated. Since circadian rhythm is apparently normal in Cnnm2 mice, is renin production normal in these mice?

7. Regarding the mechanisms underlying circadian blood pressure control it is really strange that the authors have not considered glucocorticoids and activation of the sympathetic nervous system. For example, what happens to cortisone and catecholamine levels in the Trpm6-deficient mice?

8. To fully show the importance of Mg2+ in the blood pressure changes in the Trpm6-deficient mice, it would be important to supplement the mice to assess whether Mg2+ replacement normalizes blood pressure control. These are important studies that should be considered.

9. Mg2+ has been associated with regulation of clock genes. It would be interesting to consider this in the present study where circadian BP control is altered.

10. Trpm6 is associated with Trpm7 and some studies have suggested that these Mg2+ transporters dimerize for activation. What is the status of Trpm7 in the Trpm6-/- mice?

11. Trpm7 deficiency and associated hypomagnesemia have been associated with hypertension. This should be discussed in the paper.

12. Please add the molecular size of bands in the western blot images.

Reviewer #3 (Remarks to the Author):

Funato Y. and co-workers describe the molecular mechanism associated with the reduction of blood pressure in CNNM2 deficiency, stating a role for TRPM6 in the circadian secretion of renin and thus blood pressure regulation. The authors show that Cnnm2 deficient mice have downregulation of TRPM6, and that the latter is necessary to increase renin secretion in response to -adrenergic stimulus during the active period. Using MDCT cells and siRNA-mediated silencing for Cnnm2, the authors demonstrate how Mg2+ and Cnnm2 expression regulate Trpm6 levels. In addition, altered expression of Trpm6 demonstrates to result in the loss of blood pressure circadian pattern, where the authors propose that it may be due to the lack of -adrenergic response from renin-producing cells. The study is highly relevant for the role of Mg2+ channels in blood pressure regulation. However, some concerns remain regarding the link of TRPM6 in the DCT and the signaling mechanisms that lead to renin secretion.

1. The authors indicate based on figure 2a (middle) that Cnnm2 knockdown increases intracellular Mg2+ levels, and that this was further elevated by the simultaneous knockdown of Cnnm4. However, no statistical difference is described between Cnnm2-siRNA and Cnnm2-siRNA + Cnnm4-siRNA. If there is a statistically significant difference, it should be stated in the figure. Otherwise, it is suggested to rephrase the text. Similar to this, in figure 2a (right), it should be described if there is a statistically significant difference between Cnnm2-siRNA and Cnnm2-siRNA + Cnnm4-siRNA.

2. Although some of the probably responsible molecular players are mentioned in the text (-AR,

cAMP, TRPM6), the proposed events would be taking place in adjacent, bur separate cells (figure 5). Therefore, how could the TRPM6 deficiency, or Mg2+ levels in the DCT, modulate the signaling cascade of -AR cAMP renin release, in juxtaglomerular cells. The authors should include mechanistic data and discuss the potential mechanisms (e.g. paracrine signaling, gap junctions) that could explain these results.

3. Based on figure 2b and Cnnm2/Cnnm4-siRNA in MDCT cells, the authors state that decreasing Mg2+ concentrations have a suppressive role on the expression of Trpm6 up to 0.2mM, and further reductions cancelled this effect. For this, statistical analysis from 0.1mM Mg2+ should be described in the figure and text.

4. Regarding the proposed suppressive effect of Mg2+ concentrations on the expression of Trpm6 described in figure 2b, it is important to measure intracellular levels of magnesium as a control to strengthen the hypothesis and also to provide convincing evidence that the low concentrations of extracellular Mg2+ (0.1 and 0.02 mM) lead to low levels of intracellular Mg2+, and therefore to a loss of the suppressive effect over Trpm6.

5. The authors, based on figure 5 and 6, state that -AR signaling is abrogated in JG cells from Trpm6-deficient mice, and that this is consistent with the disappearance of the circadian increase in blood pressure and renin activity. However, this statement is in contrast with previous reports from Soo Mi Kim et al., 2008, where it was shown that circadian variation in arterial blood pressure persisted in 1/2-AR-deficient mice. How could the authors explain this discrepancy?

Minor issues
1. Introduction- In line 64 and 81 the DCT is referred to as part of the “urinary duct”, please change to the correct terminology.
2. Materials and methods-Hormone quantitation: ‘...renin assay kit (Abacam)...’ has a typo: Abacam > Abcam
3. Line 373 describes IP + immunoblotting for TRPM6, however the legend of figure 1.b states that IP + immunoblotting was only done for CNNM2 and immunoblotting for the rest (NCC, pNCC, TRPM6). Authors should clarify precisely for which protein was IP + immunoblotting performed, and for which only immunoblotting, both in the manuscript and figure legend.
4. Results-line 222-223 mentions eNOS, but it is previously described as nNOS, please change or clarify.
5. Figure 2b states that p values were determined by 1-way ANOVA. However, line 482 described that these were obtained b y 2-way ANOVA. Please clarify which statistical test was performed.
6. Figure 3b legend states ‘... immunoblotting with an anti-TRPM6 antibody’. Following the description of line 373, these should be IP + immunoblotting. Please clarify.
7. Figure 4b legend mentions ‘... 14 ZT (night)...’, please change to ‘dark’. Also, reference to light and dark columns should be incorporated in the legend instead of the graph.
8. Figure 5a legend mentions Cnnm2 +/+;Six2-Cre mice, but results line 216 and figure 5a states Trpm6fl/fl;Six2-Cre mice. Please clarify.
9. Figure 6b legend mentions ‘... (Iso)’, please define the abbreviation in line 234 from the text.

Reviewer #4 (Remarks to the Author):

In the present paper, Funato and colleagues report intriguing results on the role of the TRPM6 magnesium channel in the generation of circadian oscillations in blood pressure (BP). Of note, the molecular mechanism(s) responsible for the elevation of BP at the onset of the active phase and for the dip in BP at the end of the active phase have been extensively studied by a number of laboratories. The general consensus is that this mechanism is highly complex and multifactorial. Here, Funato et al. show that disruption of the TRPM6 in a single cell type (DCT cells) is sufficient to abolish the circadian rhythm in BP.

Major

All along the manuscript the authors point on the role of the sympathetic nervous system in the

control of renin secretion and, in the elevation of the blood pressure (BP) at the onset of the activity phase. However, it is well established that renal innervation plays a relatively secondary role in BP control as compare to the tubulo-glomerular feedback (TGF) mechanism and myogenic response of the afferent arteriole. For instance, renal denervation in human and rats does not result in the loss or impairment of circadian rhythm in BP (Katayama et al. 2013; Kario et al. 2018; de la Sierra et al. 2017; Becker et al. 2017). Thus, in the first place, the authors must check
- whether renal denervation in control mice would result in the loss of the diurnal increase in BP. (n.b. – the renal denervation experiments have been performed in mice, but post-denervation circadian profile of BP is not available, to our knowledge, for this species).

The authors also state that BP is directly regulated by renin levels. Even though, this fact is well established in human and rats, there are many data suggesting that this is only partially true in mice. Indeed, in a classical scheme, renin is rate-limiting for AngII formation. However, in mice there is a dramatic difference between plasma renin activity (PRA) and plasma renin concentration (PRC) suggesting that the availability of angiotensinogen could be limiting as well, - see an excellent review on this subject in “Molecular and genetic basis of renal disease” page 23, by Mount DB and Pollak MR and, (Kim et al. 2002). Accordingly, the power of the study could be significantly improved by the inclusion of dosage of plasma:
- angiotensinogen
- angiotensin II
- aldosterone

A clear conclusion about the role of TRPM6 in circadian regulation of renin secretion and BP control cannot be formulated without a true circadian analysis of plasma renin activity as well as plasma angiotensinogen, angiotensin II and aldosterone levels.
- i.e. the dosage of renin activity and aforementioned hormones must be performed at, at least, six different time points throughout the circadian cycle (e.g. ZT0, 4, 8, 12, 16 and 20).

- blood pressure records shown in Figure 4A are totally abnormal in terms of absolute values – both systolic and diastolic pressures are at least 20 mmHg higher than the standard BP values described for C57BL6 mice (max values are: systolic at 150 mmHg and diastolic at 120 mmHg !!!). Do the authors have any explanation for these strange results? Have they checked for the second renin gene present in some mouse strains?

- The discussion does not provide any hints for the possible mechanism of interference between magnesium transport in the DCT cells and the function of the juxtaglomerular apparatus.

Minor:

- the Six2-Cre system is not commonly used for creation of kidney-specific knockout models. Accordingly, a more detailed analysis of Six2 driven Cre expression in different tissues must be provided. This is especially true for this project because the Trpm6-null mice die embryonically from multi-organ failure. For instance, Chubanov et al (Chubanov et al. 2016) have shown that TRPM6 is important in the intestine – this organ has not been tested for TRPM6 expression in the analysis presented in Figure 3A.

Becker, B. K., A. C. Feagans, D. Chen, M. Kasztan, C. Jin, J. S. Speed, J. S. Pollock, and D. M. Pollock. 2017. 'Renal denervation attenuates hypertension but not salt sensitivity in ETB receptor-deficient rats', Am J Physiol Regul Integr Comp Physiol, 313: R425-r37.

Chubanov, V., S. Ferioli, A. Wisnowsky, D. G. Simmons, C. Leitzinger, C. Einer, W. Jonas, Y. Shymkiv, H. Bartsch, A. Braun, B. Akdogan, L. Mittermeier, L. Sytik, F. Torben, V. Jurinovic, E. P.

van der Vorst, C. Weber, O. A. Yildirim, K. Sotlar, A. Schurmann, S. Zierler, H. Zischka, A. G. Ryazanov, and T. Gudermann. 2016. 'Epithelial magnesium transport by TRPM6 is essential for prenatal development and adult survival', elife, 5.

de la Sierra, A., J. Pareja, P. Armario, A. Barrera, S. Yun, S. Vazquez, L. Sans, J. Pascual, and A. Oliveras. 2017. 'Renal Denervation vs. Spironolactone in Resistant Hypertension: Effects on Circadian Patterns and Blood Pressure Variability', Am J Hypertens, 30: 37-41.

Kario, K., M. Bohm, F. Mahfoud, R. R. Townsend, M. A. Weber, M. Patel, C. C. Tyson, J. Weil, T. Agdirlioglu, S. A. Cohen, M. Fahy, and D. E. Kandzari. 2018. 'Twenty-Four-Hour Ambulatory Blood Pressure Reduction Patterns After Renal Denervation in the SPYRAL HTN-OFF MED Trial', Circulation, 138: 1602-04.

Katayama, T., D. Sueta, K. Kataoka, Y. Hasegawa, N. Koibuchi, K. Toyama, K. Uekawa, M. Mingjie, T. Nakagawa, M. Maeda, H. Ogawa, and S. Kim-Mitsuyama. 2013. 'Long-term renal denervation normalizes disrupted blood pressure circadian rhythm and ameliorates cardiovascular injury in a rat model of metabolic syndrome', J Am Heart Assoc, 2: e000197.

Kim, H. S., G. Lee, S. W. John, N. Maeda, and O. Smithies. 2002. 'Molecular phenotyping for analyzing subtle genetic effects in mice: application to an angiotensinogen gene titration', Proc Natl Acad Sci U S A, 99: 4602-7.

Point-by-point response to the reviewers' comments

To Reviewer #1

The manuscript by Funato et al. reports that conditional deletion of trpm6 in the kidney is associated with hypotension and the absence of circadian blood pressure cycling. From the results of further experiments the authors speculate, that the absence of diurnal blood presure changes, in particular, the increase of blood blood pressure during the dark phase result from an impaired beta-adrenergic stimulability of renin secretion from kidneys with conditional trpm6 deletion.
Although I wish to appreciate the successful generation of a conditional renal trpm6 knockout, I have major concerns regarding the other findings and speculations.
Response: We thank the reviewer for positive consideration on our manuscript. As described below in a point-by-point manner, we have performed multiple experiments and revised the manuscript to answer to all the reviewer's comments.

demonstration of one diurnal blood pressure cycle is not sufficient. Recordings for 5-7 subsequent days should be shown to allow an impression about the constancy of differences between the two genotypes
Response: As requested by the reviewer, we showed recording data of blood pressure for 5 consecutive days (Supplementary Figure 4), which clearly indicate the constant loss of the circadian blood pressure elevation in *Trpm6*-deficient mice.

the speculation that the almost complete absence of circadian blood pressure changes could be the result of impaired renin secretion appears somewhat far fetched. I wonder if inhibition of the RAS by ACE-inhibitors or sartans really abolishes blood pressure cycles. Therefore the authors should support their speculations by demonstrating the effects of RAS-inhibitors on blood pressure cycles.
Response: As requested by the reviewer, we tested the effect of the administration of aliskiren, a renin inhibitor in clinical use (Nat. Rev. Drug Discov. 7, 399-410). The results showed that it substantially suppressed blood pressure elevation during the active period and almost abolished the circadian changes of blood pressure (Figure 6a).

the method used to measure renin secretion from the kidney (fig.6) is really not state of the art. It is strongly recommended to determine renin secretion either from isolated perfused kidney or in vivo after injection or infusion of catecholamines. In this context the concentration of the drugs used must be given.
Response: We agree to the reviewer that it is important to measure renin levels secreted from the kidney by appropriate stimuli *in vivo*. Therefore, we injected 10 mg/kg bw isoproterenol to each mouse, according to the previous reports (Hypertension 57, 460-468; Am J Physiol Renal Physiol, 292, F415-F422), and determined blood renin levels at 30 min after the injection. The results showed that the increase was greatly suppressed in *Trpm6*-deficient mice (Figure 4c), consistent with our previous results of the renin-staining experiments using the slice preparations of the kidney.

if it would turn out that beta-adrenergic signaling in renin secreting cells of trpm6 ko kidneys is impaired it should be examined if this is a renin cell specific effect or holds for all beta-1 adrenergic receptors (for example heart rate after catecholamine administration
Response: As described above, we observed that renin secretion stimulated by isoproterenol was clearly impaired in *Trpm6*-deficient mice. Therefore, we also investigated the possible effect of isoproterenol administration on heart rate by telemetry experiments and observed no significant difference (Supplementary Figure 7), suggesting that the effect of *Trpm6*-knockout is specific to renin secretion in the kidney.

it should be checked, if the possible effects of trpm6 deletion on blood pressure and on beta-adrenergic signaling can be compensated by magnesium supplementation
Response: We thank the reviewer for pointing out the importance of magnesium homeostasis in blood pressure regulation. We tested the effect of feeding wild-type mice with high magnesium

diet containing 0.6% magnesium (normal diet: 0.3% magnesium). The results showed that it substantially reduced blood pressure elevation during the active period and the circadian changes almost disappeared (Figure 6c), as observed in *Trpm6*-deficient mice. Such a strong effect of supplemental magnesium on blood pressure is remarkable, but consistent with its suppressive effect on *Trpm6* expression observed in mice fed high magnesium diet (Supplementary Figure 8).

To Reviewer #2

This study by Funato et al investigated the role of TRPM6 in blood pressure regulation by studying kidney-specific Trpm6-deficient mice. Results demonstrate that Trpm6-deficient mice have reduced blood pressure and a blunted circadian rhythm. This was associated with reduced agonist-stimulated renin secretion in kidney sections. Based on these findings the authors conclude that TRPM6 plays a critical role in diurnal blood pressure regulation and this involves renin. This is an interesting study that combines mouse models and in vitro studies. However there are a number of aspects that warrant further consideration.

Response: We thank the reviewer for positive consideration on our manuscript. As described below in a point-by-point manner, we have performed multiple experiments and revised the manuscript to answer to all the reviewer's comments.

1. This study builds on previous findings by the group where it was shown that in Cnnm2-deficient mice, renal Trpm6 expression is reduced with associated decrease in blood pressure but normal circadian rhythm. Here it is shown the Trpm6-deficient mice have reduced blood pressure with loss of circadian rhythm. In both conditions Trpm6 is reduced with decreased Mg2+, hence the altered circadian rhythm may be due to factors other than renal Trpm6 downregulation and hypomagnesemia. Please discuss this in the context of the present studies.

2. The studies relating to Cnnm2 (figures 1 and 2) do not contribute significantly to the overall goals of the present study. Also, it is unclear exactly how Cnnm2 and Trpm6 interact in the kidney and whether their interaction is simply secondary to changes in intracellular Mg. Please expand.

Response: As pointed out by the reviewer, *Cnnm2*-deficient mice and *Trpm6*-deficient mice commonly have lowered blood pressure, but the details of the circadian variation are qualitatively different. We think that *Trpm6* expression levels should be important for the phenotypical difference: *Trpm6* expression was almost completely suppressed in *Trpm6*-deficient mice, but approximately half remained in *Cnnm2*-deficient mice. However, we must admit that the phenotype of *Cnnm2*-deficient mice cannot be explained by the observed decrease of *Trpm6* expression. To make the point clearer, we moved the data of *Cnnm2*-deficient mice and MDCT culture cells (Figures 1 and 2 in the original version) to supplemental information (Supplementary Figure 1) and shortened the description about them. Furthermore, we added a discussion about this point by explicitly stating the qualitative difference between *Cnnm2*-deficient mice and *Trpm6*-deficient mice (page 15, line 20 – page 16, line 7).

3. A major finding in this study is the lack of circadian rhythm in the Trpm6-deficient mice. However it should be noted that the blood pressures in the wild type mice are high, reaching levels levels as high as 160/120 mmHg during the dark periods (figure 4a, 4b). These pressures, measured by telemetry are pathological and by definition, the mice would be considered to be hypertensive. Moreover, even at baseline (time 0), blood pressures seem high (normal blood pressure in mice is about 110/80 mmHg, and may be lower with telemetry readings). Hence, there are concerns regarding the Trpm6+/+ control mice.

Response: We thank the reviewer for pointing out this very important problem. We realized that the blood pressure levels were abnormally high. In the previous experiments, we measured blood pressure levels 2 weeks after implanting the telemetry probe. We thought that the mice had not yet recovered sufficiently from the surgical damage and they showed abnormally high blood pressure. Therefore, in this revision, mice were kept acclimated for 3 weeks after

implantation of the probe, and then blood pressure was measured. The results showed that 24 h blood pressure of control mice was 118/93 mmHg, which is considered to be in the normal range (120 ± 2/99 ± 2; Am. J. Hypertens. 14, 405-408). Moreover, we confirmed the significant decrease of blood pressure and the disappearance of the circadian upsurge during the active period in *Trpm6*-deficient mice, as in the previous experiments. We re-performed all the experiments measuring blood pressure under the condition with 3 week acclimation and replaced the data (Figures 2 and 6).

4. The studies related to kidney renin production in basal and stimulated states are superficial and are based on qualitative (at most semi-quantitative) methods using immunofluorescence. This is sub-optimal because major conclusions are made in this study relating to renin.
5. As stated above, all the renin studies are qualitative based on immunofluorescence approaches (figs 5, 6). More specific assays for renin measurement in tissues/cells need to be included. This is critical considering the conclusions of the study.
Response: We agree that the renin-related experiments in the previous version were not sufficient to make a definitive conclusion. Therefore, we performed additional experiments to directly measure renin levels secreted from the kidney by appropriate stimuli *in vivo*. We injected isoproterenol to live mice and determined secreted renin levels in the blood at 30 min after the injection. The results showed that the increase was greatly suppressed in *Trpm6*-deficient mice (Figure 4c), consistent with our previous results of the renin-staining experiments using the slice preparations of the kidney.

6. It would be useful to know what happens to renin production in kidneys from Cnnm2 mice, in which Trpm6 is downregulated. Since circadian rhythm is apparently normal in Cnnm2 mice, is renin production normal in these mice?
Response: As requested by the reviewer, we also checked blood renin levels in *Cnnm2*-deficient mice and confirmed that the circadian variation was preserved (Supplementary Figure 9).

7. Regarding the mechanisms underlying circadian blood pressure control it is really strange that the authors have not considered glucocorticoids and activation of the sympathetic nervous system. For example, what happens to cortisone and catecholamine levels in the Trpm6-deficient mice?
Response: As requested by the reviewer, we examined blood noradrenaline levels in *Trpm6*-deficient mice and found that their circadian variation also disappeared as renin. It has been reported in several papers that RAS activation stimulates the release of noradrenaline to the bloodstream (Eur. J. Pharmacol. 52, 375-377; Am. J. Physiol. Heart Circ. Physiol. 281, H813-H822), and thus, it seems reasonable that both renin and noradrenaline levels showed similar results. We included the results in Figure 2e.

It should be noted that while the two are assumed to be in a positive feedback relationship, which stimulates each other's secretion, the results of isoproterenol administration experiments (Figure 4) suggest that the abnormality in renin secretion is the primary cause, affecting blood noradrenaline levels in a secondary manner, as we originally concluded. We also included these descriptions in discussion (page 18, lines 11–20).

8. To fully show the importance of Mg2+ in the blood pressure changes in the Trpm6-deficient mice, it would be important to supplement the mice to assess whether Mg2+ replacement normalizes blood pressure control. These are important studies that should be considered.
Response: We thank the reviewer for pointing out the importance of magnesium homeostasis in blood pressure regulation. We tested the effect of feeding wild-type mice with high magnesium diet containing 0.6% magnesium (normal diet: 0.3% magnesium). The results showed that it substantially reduced blood pressure elevation during the active period and the circadian changes almost disappeared (Figure 6c), as observed in *Trpm6*-deficient mice. Such a strong effect of supplemental magnesium on blood pressure is remarkable, but consistent with its

suppressive effect on *Trpm6* expression observed in these high magnesium diet-fed mice (Supplementary Figure 8, more drastic decrease than renal *Cnnm2* ablation).

9. Mg2+ has been associated with regulation of clock genes. It would be interesting to consider this in the present study where circadian BP control is altered.
Response: As pointed out by the reviewer, Mg^{2+} itself has been associated with the regulation of circadian rhythm (Nature 532, 375-379; JCI Insight 2, e91722). We checked the expression of *Bmal1* and *Per2*, well-known circadian clock genes, and found no abnormalities in their rhythmic expression patterns in *Trpm6*-deficient mice (Supplementary Figure 5). Taken together with the previous results showing no abnormalities in locomotive activity and AVP expression, the effect of *Trpm6*-knockout is considered to be specific to blood pressure regulation and does not affect the general function of the circadian clock system in the body.

10. Trpm6 is associated with Trpm7 and some studies have suggested that these Mg2+ transporters dimerize for activation. What is the status of Trpm7 in the Trpm6-/- mice?
Response: As requested by the reviewer, we examined *Trpm7* expression levels by quantitative PCR and found no significant alterations in *Trpm6*-deficient mice (Supplementary Figure 5).

11. Trpm7 deficiency and associated hypomagnesemia have been associated with hypertension. This should be discussed in the paper.
Response: As requested by the reviewer, we added a brief discussion about the relationship between TRPM7 and hypertension in the revised manuscript (page 15, lines 6–15).

12. Please add the molecular size of bands in the western blot images.
Response: We thank the reviewer for pointing out these careless problems. We added size markers to all the western blot panels.

To Reviewer #3

Funato Y. and co-workers describe the molecular mechanism associated with the reduction of blood pressure in CNNM2 deficiency, stating a role for TRPM6 in the circadian secretion of renin and thus blood pressure regulation. The authors show that Cnnm2 deficient mice have downregulation of TRPM6, and that the latter is necessary to increase renin secretion in response to -adrenergic stimulus during the active period. Using MDCT cells and siRNA-mediated silencing for Cnnm2, the authors demonstrate how Mg2+ and Cnnm2 expression regulate Trpm6 levels. In addition, altered expression of Trpm6 demonstrates to result in the loss of blood pressure circadian pattern, where the authors propose that it may be due to the lack of -adrenergic response from renin-producing cells. The study is highly relevant for the role of Mg2+ channels in blood pressure regulation. However, some concerns remain regarding the link of TRPM6 in the DCT and the signaling mechanisms that lead to renin secretion.
Response: We thank the reviewer for positive consideration on our manuscript. We understand the reviewer's concern regarding the link between TRPM6 knockout in DCT cells and renin secretion defects in JG cells. As described in our reply to the 2nd comment, we added new data on the expression of β-AR and discussions about the linking mechanism.

1. The authors indicate based on figure 2a (middle) that Cnnm2 knockdown increases intracellular Mg2+ levels, and that this was further elevated by the simultaneous knockdown of Cnnm4. However, no statistical difference is described between Cnnm2-siRNA and Cnnm2-siRNA + Cnnm4-siRNA. If there is a statistically significant difference, it should be stated in the figure. Otherwise, it is suggested to rephrase the text. Similar to this, in figure 2a (right), it should be described if there is a statistically significant difference between Cnnm2-siRNA and Cnnm2-siRNA + Cnnm4-siRNA.
Response: We rechecked the result of the statistical analyses between *Cnnm2*-siRNA and *Cnnm2*-siRNA + *Cnnm4*-siRNA, and found that there was a significant difference in *Trpm6* expression, but not in intracellular Mg^{2+} levels. Thus, we rephrased the text to avoid misleading readers as if there was a significant difference in intracellular Mg^{2+} levels (page 7, lines 8–9).

2. Although some of the probably responsible molecular players are mentioned in the text (-AR, cAMP, TRPM6), the proposed events would be taking place in adjacent, bur separate cells (figure 5). Therefore, how could the TRPM6 deficiency, or Mg2+ levels in the DCT, modulate the signaling cascade of -AR cAMP renin release, in juxtaglomerular cells. The authors should include mechanistic data and discuss the potential mechanisms (e.g. paracrine signaling, gap junctions) that could explain these results.

Response: We also consider this is a very important point that needs to be explained in more detail. We added a discussion about the potential mechanisms of intercellular communication between DCT and JG cells, by referring to the current reports about paracrine signaling and gap junctions of JG cells (page 17, line 10 – page 18, line 10). The revised discussion is also based on our additional data showing that the downregulation of AR in JG cells is not just a speculation but actually occurs in *Trpm6*-knockout mice. Besides immunofluorescence staining, we quantitatively analyzed the expression level of AR in JG cells by performing JG cell isolation by Percoll density gradient centrifugation and subsequent immunoblotting. These results explicitly indicate that the expression of AR in JG cells decreased, which can clearly explain the phenotype in the abnormal renin secretion by *Trpm6* deficiency and is also informative to discuss the potential ways for DCT-JG cell communication. We included these data in Figure 5 in the revised manuscript.

3. Based on figure 2b and Cnnm2/Cnnm4-siRNA in MDCT cells, the authors state that decreasing Mg2+ concentrations have a suppressive role on the expression of Trpm6 up to 0.2mM, and further reductions cancelled this effect. For this, statistical analysis from 0.1mM Mg2+ should be described in the figure and text.

Response: There was no significant difference in *Trpm6* expression under 0.1 mM Mg^{2+} condition, and we added the description in the figure (Supplementary Figure 1h) and text (page 7, lines 19–21).

4. Regarding the proposed suppressive effect of Mg2+ concentrations on the expression of Trpm6 described in figure 2b, it is important to measure intracellular levels of magnesium as a control to strengthen the hypothesis and also to provide convincing evidence that the low concentrations of extracellular Mg2+ (0.1 and 0.02 mM) lead to low levels of intracellular Mg2+, and therefore to a loss of the suppressive effect over Trpm6.

Response: As requested by the reviewer, we measured $[Mg^{2+}]_i$ of cells cultured under various Mg^{2+} concentrations. As expected, decreasing extracellular Mg^{2+} concentrations led to the decrease of intracellular Mg^{2+} levels and narrowed the gap between control and *Cnnm2/4* knockdown cells, which can explain why the suppressive effect of *Cnnm2/4* knockdown over *Trpm6* expression was lost by lowering extracellular Mg^{2+} levels. We included these data in Supplementary Figure 1h in the revised manuscript.

5. The authors, based on figure 5 and 6, state that -AR signaling is abrogated in JG cells from Trpm6-deficient mice, and that this is consistent with the disappearance of the circadian increase in blood pressure and renin activity. However, this statement is in contrast with previous reports from Soo Mi Kim et al., 2008, where it was shown that circadian variation in arterial blood pressure persisted in 1/2-AR-deficient mice. How could the authors explain this discrepancy?

Response: As the reviewer pointed out, Soo Mi Kim et al. claim that circadian variation remained in 1/2-AR-deficient mice. However, it should be mentioned that the amplitude of blood pressure variation was apparently smaller (approximately half) in their 1/2-AR-deficient mice, and they ascribed it to the decreased locomotor activity. Furthermore, the unusual background of their mice should also be considered carefully; while we used pure C57BL6/J background mice, they used mixed background of three stains (C57BL6/J, 129, and FVB) since 1/2-AR-deficient mice in pure C57BL6/J background were lethal. In rats, it is well known that genetic backgrounds are critically important factors for circadian blood pressure variation (J. Hypertens. 22, 727-737), and thus, it is possible that these background differences may alter the

impact of decreased AR expression. Collectively, their paper should have been referred and discussed in the original manuscript, but it does not necessarily rule out our model that the decreased AR expression in JG cells leads to the blunted blood pressure variation. We briefly explained these points in discussion (page 17, lines 2–9).

Minor issues
1. Introduction- In line 64 and 81 the DCT is referred to as part of the "urinary duct", please change to the correct terminology.
2. Materials and methods-Hormone quantitation: '...renin assay kit (Abacam)...' has a typo: Abacam > Abcam
3. Line 373 describes IP + immunoblotting for TRPM6, however the legend of figure 1.b states that IP + immunoblotting was only done for CNNM2 and immunoblotting for the rest (NCC, pNCC, TRPM6). Authors should clarify precisely for which protein was IP + immunoblotting performed, and for which only immunoblotting, both in the manuscript and figure legend.
4. Results-line 222-223 mentions eNOS, but it is previously described as nNOS, please change or clarify.
5. Figure 2b states that p values were determined by 1-way ANOVA. However, line 482 described that these were obtained by 2-way ANOVA. Please clarify which statistical test was performed.
6. Figure 3b legend states '… immunoblotting with an anti-TRPM6 antibody'. Following the description of line 373, these should be IP + immunoblotting. Please clarify.
7. Figure 4b legend mentions '… 14 ZT (night)…', please change to 'dark'. Also, reference to light and dark columns should be incorporated in the legend instead of the graph.
8. Figure 5a legend mentions Cnnm2 +/+;Six2-Cre mice, but results line 216 and figure 5a states Trpm6fl/fl;Six2-Cre mice. Please clarify.
9. Figure 6b legend mentions '… (Iso)', please define the abbreviation in line 234 from the text.
Response: We thank the reviewer for carefully reading the manuscript and pointing out these mistakes. We corrected the text and legends according to each comment, except for comments 6 and 7. For comment 6, the result of Fig. 3a in the original manuscript (Fig. 1a in the revised manuscript) was obtained by direct immunoblotting using commercial anti-TRPM6 antibody. IP + immunoblotting was performed only when we used our house made anti-TRPM6 antibody, in order to validate its specificity (Supplementary Fig. 10). We added some description in the methods section and figure legend to make the point clear (page 22, lines 3–4 and Supplementary Fig. 10). For comment 7, we replaced the data with those from six different time points in response to the comment raised by another reviewer, and thus, we indicate each time point and the region of light/dark time periods on the horizontal axis, as in other panels (Figure 2, c and d).

To Reviewer #4

In the present paper, Funato and colleagues report intriguing results on the role of the TRPM6 magnesium channel in the generation of circadian oscillations in blood pressure (BP). Of note, the molecular mechanism(s) responsible for the elevation of BP at the onset of the active phase and for the dip in BP at the end of the active phase have been extensively studied by a number of laboratories. The general consensus is that this mechanism is highly complex and multifactorial. Here, Funato et al. show that disruption of the TRPM6 in a single cell type (DCT cells) is sufficient to abolish the circadian rhythm in BP.
Response: We thank the reviewer for indicating the important and interesting point of our study. To emphasize the functional importance of TRPM6 in DCT cells, we added mechanistic discussions about the link between DCT cells and renin-secreting cells (page 17, line 10 - page 18, line 10).

All along the manuscript the authors point on the role of the sympathetic nervous system in the control of renin secretion and, in the elevation of the blood pressure (BP) at the onset of the activity phase. However, it is well established that renal innervation plays a relatively secondary role in BP control as

compare to the tubulo-glomerular feedback (TGF) mechanism and myogenic response of the afferent arteriole. For instance, renal denervation in human and rats does not result in the loss or impairment of circadian rhythm in BP (Katayama et al. 2013; Kario et al. 2018; de la Sierra et al. 2017; Becker et al. 2017). Thus, in the first place, the authors must check

- whether renal denervation in control mice would result in the loss of the diurnal increase in BP. (n.b. – the renal denervation experiments have been performed in mice, but post-denervation circadian profile of BP is not available, to our knowledge, for this species).

Response: We agree that the suggested point is very important, and thus, we performed renal denervation experiments to our mice. As shown in Figure 6b, the circadian variation of blood pressure disappeared by renal denervation, which supports our model.

As for the indicated studies in human and rats, it is true that all failed to find the effect of renal denervation on circadian variation in blood pressure, but we consider that these studies do not necessarily deny our model. The rat experiments were performed under conditions impossible to see the effect on circadian variation in the first place; denervation operations were done on non-dipper hypertensive strains (Katayama et al.) or on animals with almost no circadian variation even in sham groups (Becker et al., amplitude less than 2 mmHg). As for denervation on human patients, there are other studies showing reduced circadian variation in blood pressure (Lancet 391, 2335-2345), and the effect of renal denervation operations is known to heavily depend on methods and patients selected. Collectively, the situation is so complicated that we discussed about these rat and human studies by citing the abovementioned papers (page 19, lines 6–16).

The authors also state that BP is directly regulated by renin levels. Even though, this fact is well established in human and rats, there are many data suggesting that this is only partially true in mice. Indeed, in a classical scheme, renin is rate-limiting for AngII formation. However, in mice there is a dramatic difference between plasma renin activity (PRA) and plasma renin concentration (PRC) suggesting that the availability of angiotensinogen could be limiting as well, - see an excellent review on this subject in "Molecular and genetic basis of renal disease" page 23, by Mount DB and Pollak MR and, (Kim et al. 2002). Accordingly, the power of the study could be significantly improved by the inclusion of dosage of plasma:

- angiotensinogen

- angiotensin II

- aldosterone

Response: We thank the reviewer for pointing out the importance of the species difference. We additionally analyzed the level of angiotensinogen, the other rate limiting molecule for AngII formation. We found that unlike renin, angiotensinogen levels were not affected by *Trpm6* knockout, which is consistent with our original conclusion that the abnormality in renin secretion itself is the primary cause of blunted circadian blood pressure variation by *Trpm6* knockout. We added the data in Supplementary Figure 6.

A clear conclusion about the role of TRPM6 in circadian regulation of renin secretion and BP control cannot be formulated without a true circadian analysis of plasma renin activity as well as plasma angiotensinogen, angiotensin II and aldosterone levels.

- i.e. the dosage of renin activity and aforementioned hormones must be performed at, at least, six different time points throughout the circadian cycle (e.g. ZT0, 4, 8, 12, 16 and 20).

Response: As requested by the reviewer, we analyzed renin activity (and angiotensinogen) at six different time points during one day. We found that renin activity in control mice showed a clear circadian variation, which is consistent with our original observation (peaking at ZT14). By contrast, such circadian variation of renin activity was not observed in *Trpm6*-knockout mice, confirming our original conclusion. We showed the results in Figure 2d in the revised manuscript.

- blood pressure records shown in Figure 4A are totally abnormal in terms of absolute values – both systolic and diastolic pressures are at least 20 mmHg higher than the standard BP values described for

C57BL6 mice (max values are: systolic at 150 mmHg and diastolic at 120 mmHg !!!). Do the authors have any explanation for these strange results? Have they checked for the second renin gene present in some mouse strains?

Response: We thank the reviewer for pointing out this very important problem. We realized that the blood pressure levels were abnormally high. In the previous experiments, we measured blood pressure levels 2 weeks after implanting the telemetry probe. We thought that the mice had not yet recovered sufficiently from the surgical damage and they showed abnormally high blood pressure. Therefore, in this revision, mice were kept acclimated for 3 weeks after implantation of the probe, and then blood pressure was measured. The results showed that max blood pressure of control mice was 130/103 mmHg, which is considered to be in the normal range (max 138/109; The mouse in Biomedical Research, 2 nd Ed.). Moreover, we confirmed the significant decrease of blood pressure and the disappearance of the circadian upsurge during the active period in *Trpm6*-deficient mice, as in the previous experiments. We re-performed all the experiments measuring blood pressure under the condition with 3 week acclimation and replaced the data (Figures 2 and 6).

- The discussion does not provide any hints for the possible mechanism of interference between magnesium transport in the DCT cells and the function of the juxtaglomerular apparatus.

Response: We thank the reviewer for pointing out this problem. We should admit that the original version may mislead readers that magnesium transport in DCT cells, the common function of CNNM2 and TRPM6, is important. It should be noted that we consider magnesium transport in DCT itself is not directly involved in renin secretion from JG cells; magnesium transport in DCT is abolished by either *Cnnm2* or *Trpm6* deficiency, but the phenotype of *Cnnm2* and *Trpm6* deficient mice on blood pressure are qualitatively different (only *Trpm6* deficient mice showed disappeared circadian variation). Therefore, the expression of TRPM6 (and its functions besides magnesium transport) seems to be crucial for the regulation of circadian renin release from JG cells. We added a discussion about the abovementioned point and the potential mechanisms of how loss of TRPM6 affects the status of adrenergic receptor in the adjacent JG cells (page 17, line 10 – page 18, line 10). Moreover, to avoid the misunderstanding that magnesium transport in DCT itself is important, we moved the results from *Cnnm2*-deficient mice to Supplementary Figure 1 and simplified the explanation.

Minor:

- the Six2-Cre system is not commonly used for creation of kidney-specific knockout models. Accordingly, a more detailed analysis of Six2 driven Cre expression in different tissues must be provided. This is especially true for this project because the Trpm6-null mice die embryonically from multi-organ failure. For instance, Chubanov et al (Chubanov et al. 2016) have shown that TRPM6 is important in the intestine – this organ has not been tested for TRPM6 expression in the analysis presented in Figure 3A.

Response: As requested by the reviewer, we analyzed the tissue distribution of Cre by immunoblotting. Since *Six2-Cre* mice have been reported to express GFP-Cre fusion proteins in embryonic stages (Cell Stem Cell 2, 284-291), we performed anti-GFP immunoblotting analyses with the tissue lysates obtained from E17.5 mouse embryos. The results showed that GFP-Cre is specifically expressed in the kidney but not in other organs including the intestine. We added the results in Supplementary Figure 3.

Becker, B. K., A. C. Feagans, D. Chen, M. Kasztan, C. Jin, J. S. Speed, J. S. Pollock, and D. M. Pollock. 2017. 'Renal denervation attenuates hypertension but not salt sensitivity in ETB receptor-deficient rats', Am J Physiol Regul Integr Comp Physiol, 313: R425-r37.

Chubanov, V., S. Ferioli, A. Wisnowsky, D. G. Simmons, C. Leitzinger, C. Einer, W. Jonas, Y. Shymkiv, H. Bartsch, A. Braun, B. Akdogan, L. Mittermeier, L. Sytik, F. Torben, V. Jurinovic, E. P. van der Vorst, C. Weber, O. A. Yildirim, K. Sotlar, A. Schurmann, S. Zierler, H. Zischka, A. G. Ryazanov, and T. Gudermann. 2016. 'Epithelial magnesium transport by TRPM6 is essential for prenatal development and adult survival', elife, 5.

de la Sierra, A., J. Pareja, P. Armario, A. Barrera, S. Yun, S. Vazquez, L. Sans, J. Pascual, and A. Oliveras. 2017. 'Renal Denervation vs. Spironolactone in Resistant Hypertension: Effects on Circadian Patterns and Blood Pressure Variability', Am J Hypertens, 30: 37-41.
Kario, K., M. Bohm, F. Mahfoud, R. R. Townsend, M. A. Weber, M. Patel, C. C. Tyson, J. Weil, T. Agdirlioglu, S. A. Cohen, M. Fahy, and D. E. Kandzari. 2018. 'Twenty-Four-Hour Ambulatory Blood Pressure Reduction Patterns After Renal Denervation in the SPYRAL HTN-OFF MED Trial', Circulation, 138: 1602-04.
Katayama, T., D. Sueta, K. Kataoka, Y. Hasegawa, N. Koibuchi, K. Toyama, K. Uekawa, M. Mingjie, T. Nakagawa, M. Maeda, H. Ogawa, and S. Kim-Mitsuyama. 2013. 'Long-term renal denervation normalizes disrupted blood pressure circadian rhythm and ameliorates cardiovascular injury in a rat model of metabolic syndrome', J Am Heart Assoc, 2: e000197.
Kim, H. S., G. Lee, S. W. John, N. Maeda, and O. Smithies. 2002. 'Molecular phenotyping for analyzing subtle genetic effects in mice: application to an angiotensinogen gene titration', Proc Natl Acad Sci U S A, 99: 4602-7.

Response: We thank the reviewer for the information of the related papers, which were very helpful for us to understand the details. We added most of the papers listed here in the reference in the revised manuscript.

REVIEWERS' COMMENTS

Reviewer #1 (Remarks to the Author):

The authors have satisfactorily addressed my concerns

Reviewer #2 (Remarks to the Author):

This is an interesting study that has been well conducted.
The authors have extensively revised the paper.
All my concerns have been addressed.

Reviewer #3 (Remarks to the Author):

The majority of the raised points have been adequately addressed therefore the revised manuscript is significantly approved.

Reviewer #4 (Remarks to the Author):

The authors have successfully addressed all questions and comments raised in my review.

Point-by-point response to the reviewers' comments

To Reviewer #1

The authors have satisfactorily addressed my concerns
Response: We thank the reviewer for giving us many constructive suggestions and accepting our response in the previous revision. The quality of the manuscript has been significantly improved.

To Reviewer #2

This is an interesting study that has been well conducted.
The authors have extensively revised the paper.
All my concerns have been addressed.
Response: We thank the reviewer for giving us many constructive suggestions and accepting our response in the previous revision. The quality of the manuscript has been significantly improved.

To Reviewer #3

The majority of the raised points have been adequately addressed therefore the revised manuscript is significantly approved.
Response: We thank the reviewer for giving us many constructive suggestions and accepting our response in the previous revision. The quality of the manuscript has been significantly improved.

To Reviewer #4

The authors have successfully addressed all questions and comments raised in my review.
Response: We thank the reviewer for giving us many constructive suggestions and accepting our response in the previous revision. The quality of the manuscript has been significantly improved.
